# Assessment of recommended approaches for containment and safe handling of human excreta in emergency settings

**DOI:** 10.1371/journal.pone.0201344

**Published:** 2018-07-26

**Authors:** Diogo Trajano Gomes da Silva, Edgard Dias, James Ebdon, Huw Taylor

**Affiliations:** 1 Environment and Public Health Research Group, School of Environment and Technology, University of Brighton, Brighton, United Kingdom; 2 Department of Sanitary and Environmental Engineering, Faculty of Engineering, Federal University of Juiz de Fora (UFJF), Juiz de Fora, Minas Gerais, Brazil; Médecins Sans Frontières, BELGIUM

## Abstract

Ebola and cholera treatment centres (ETC and CTC) generate considerable quantities of excreta that can further the transmission of disease amongst patients and health workers. Therefore, approaches for the safe handling, containment and removal of excreta within such settings are needed to minimise the likelihood of onward disease transmission. This study compared the performance and suitability of three chlorine-based approaches (0.5% HTH, NaDCC and NaOCl (domestic bleach)) and three lime-based approaches (10%, 20% and 30% Ca(OH)_2_). The experiments followed recent recommendations for Ebola Treatment Centres. Three excreta matrices containing either raw municipal wastewater, or raw municipal wastewater plus 10% or 20% (w/v) added faecal sludge, were treated in 14 litre buckets at a ratio of 1:10 (chlorine solutions or lime suspensions: excreta matrix). The effects of mixing versus non-mixing and increasing contact time (10 and 30 mins) were also investigated. Bacterial (faecal coliforms (FC) and intestinal enterococci (IE)) and viral (somatic coliphages (SOMPH), F^+^specific phages (F+PH) and *Bacteroides fragilis* phages (GB-124PH)) indicators were used to determine the efficacy of each approach. Lime-based approaches provided greater treatment efficacy than chlorine-based approaches, with lime (30% w/v) demonstrating the greatest efficacy (log reductions values, FC = 4.75, IE = 4.16, SOMPH = 2.85, F+PH = 5.13 and GB124PH = 5.41). There was no statistical difference in efficacy between any of the chlorine-based approaches, and the highest log reduction values were: FC = 2.90, IE = 2.36, SOMPH = 3.01, F+PH = 2.36 and GB124PH = 0.74. No statistical difference was observed with respect to contact time for any of the approaches, and no statistical differences were observed with respect to mixing for the chlorine-based approaches. However, statistically significant increases in the efficacy of some lime-based approaches were observed following mixing. These findings provide evidence and practical advice to inform safe handling and containment of excreta and ensure more effective health protection in future emergency settings.

## Introduction

The recent West Africa Ebola virus disease (EVD) outbreak (2013–2016) led to the deaths of 11,310 people (28,616 cases in total) [1]. Although the vast majority of cases occurred in West Africa, a few cases were registered in the United States and in some European countries [2]. EVD is a severe illness in humans, having an average fatality rate of around 50%. It is transmitted through direct contact with the blood, secretions, organs or other bodily fluids of infected people, and with surfaces and materials (e.g. clothing) that are contaminated with these fluids [3].

The Ebola virus (EBV), is recognised to be relatively fragile and less resistant to environmental factors than enteric viruses [4] and there are suggestions that it is not normally transmitted via the faecal-oral transmission pathway [5]. However, this assumption has been disputed, as the EBV has been isolated by cell culture from the urine of infected patients and Ebola RNA has been detected in patient stools and urine [6]. Furthermore, some studies reported that EBV and a suggested EBV surrogate (phage phi 6) can persist in wastewater for up to 8 days [7, 8]. Given the very low infectious dose associated with EBV [9], even the presence of low concentrations in wastewater and human excreta may be potentially sufficient to cause human infection. Therefore, the limited evidence to date supports a precautionary approach to the safe handling and containment of wastewater and human excreta in Ebola emergency settings.

In order to prevent on-going transmission at Ebola Treatment Centres (ETC), institutions involved with the Water, Sanitation and Hygiene (WASH) response, such as Doctors without Borders (MSF), the U.S. Centers for Disease Control and Prevention (CDC) and the World Health Organization (WHO), took precautionary actions and recommended the addition of a 0.5% (or 5,000 mg/L) chlorine solution to a bucket partially filled with human excreta [10–12]. The layer of chlorine solution covering the excreta acts as a chemical liquid cover to inspire prudence and allow as safe as possible the handling and containment of the contents of the bucket during transportation to where it is emptied.

Chlorine compounds commonly recommended for use in ETC have been powdered calcium hypochlorite (HTH); granular sodium dichloroisocyanurate (NaDCC or SDIC) and liquid sodium hypochlorite (NaOCl) (domestic bleach). For each of these compounds, treatment efficacy can vary, according to the concentration of the chlorine solution, contact time, temperature, pH level, and the presence of organic matter [13]. Previous research has shown that sodium hypochlorite solutions are capable of effectively destroying EBV (Mak variant) suspended in a simulated organic matrix and placed on PPE and stainless steel surfaces commonly found within clinical settings [14].

Chlorination is also commonly used to treat drinking water in emergency settings [15]. However, chlorine-based compounds have been shown to lose their efficacy in matrices containing large amounts of solids and dissolved organic matter (e.g., human excreta) as they react with organic matter, forming chloro-organic and chloramines with relatively low disinfecting power [16, 17]. Free chlorine is the most effective form of chlorine and recent studies [18] have demonstrated that the EBV is highly sensitive to free chlorine, but indicated that this efficacy might not be achieved in more complex matrices (e.g., human excreta) because of the associated high chlorine demand. Therefore, the safe handling and containment of pathogen-laden human excreta may be harder to ensure in such matrices. Further evidence of potential limitations associated with chlorine-based approaches in simulated human excreta matrices have also been recently described [19].

As a consequence, there remains a clear lack of information on how best to handle human excreta in emergency settings according to current protocols recommended by humanitarian agencies. In the absence of detailed information on the survival of the EBV within human excreta matrices, the WHO and UNICEF brought together a group of international experts in the field to support a critical review of existing WASH practices and to provide updated advice in light of questions raised by practitioners in the field. The resulting document–“Ebola Virus Disease (EVD): Key questions and answers concerning water, sanitation and hygiene”–suggested that a physico-chemical treatment using hydrated lime suspensions might be a potential alternative to the use of chlorine based approaches [20].

Hydrated lime, also known as slaked lime, or calcium hydroxide (Ca (OH)_2_) is a white powder obtained when calcium oxide (CaO), or lime is mixed with water, which produces a highly alkaline suspension (c. pH 14). The use of hydrated lime to treat wastewater and faecal sludge dates back to Roman times, when it was mainly used to control odours arising from pit latrines [21]. It has subsequently been observed that effective treatment of wastewater may be achieved through exposure of excreta-borne pathogens to an alkaline environment, resulting in pathogen deactivation and destruction [22, 23]. In addition to the chemical treatment processes mediated by the high-pH environment, hydrated lime may act as a coagulating agent, resulting in a coagulation-flocculation process in which pathogens adhere to solid flocs and are removed by a sedimentation stage, along with a significant portion of the organic component of the wastewater [24].

Hydrated lime has been suggested as an alternative treatment of municipal wastewater [25, 26, and 27] and studies have demonstrated the bactericidal and virucidal properties of lime in sewage [28], faeces [29], wastewater [22, 23] and sewage sludge [30, 31]. More recently, the successful application of hydrated lime to treat wastewaters from cholera treatment centres (CTC) in Haiti following the 2010 cholera outbreak has been reported [32]. Also, a recent study in Malawi demonstrated that hydrated lime may also constitute a promising faecal sludge sanitizer for emergency settings [33].

Current WASH sector recommendations for excreta clean-up, containment and removal in Ebola Treatment Centres (ETC) are summarised in S1 Table. Generally, the protocols lack detailed information on the amount and ratio of chlorine to be applied to the human excreta. There is also a paucity of data regarding the influence of mixing on the performance of chlorine and lime-based approaches, especially given that mixing occurs naturally either through the action of pouring excreta into buckets, or by the turbulent diffusion that occurs when the bucket is removed and transported a distance to where its contents can be disposed of (or treated) safely. Although the West Africa Ebola outbreak was the stimulus for this work, the objectives were broader in that the study sought to achieve a more universal evidence-based approach to the on-site handling of human excreta. As such, it was hoped that the approach would be applicable to a wide range of disaster settings in which infectious disease outbreaks are likely among densely populated communities of displaced people. This study was specifically intended to inform clean-up, containment and removal of bodily wastes from patient bed areas, rather than final disposal which would potentially include disinfection of latrines, septic tanks, holding tanks or sewerage networks.

The aim of the research described here was to determine the suitability of bucket treatments as part of intervention protocols [10–12 and 20] for the safe clean-up and removal of human excreta currently recommended for use in emergency settings. The principal objectives were: (1) to assess the suitability of the various approaches using simulated human excreta matrices containing varying levels of suspended and dissolved solids; (2) to assess the suitability of chlorine-based approaches prepared from various commercially-available chlorine products; (3) to assess the suitability of lime-based approaches using 10%, 20% and 30% (w/v) hydrated lime suspensions; and (4) to determine the effect of contact time (Ct) (10 and 30 mins) and mixing on excreta matrices.

## Material and methods

All laboratory assessments were performed at the Environment and Public Health Research Group (EPHReG) facilities at the University of Brighton (UoB), UK. Excreta matrices (EM) were produced from dewatered faecal sludge and fresh untreated municipal wastewater collected weekly from a local municipal wastewater treatment plant (Hailsham North WWTP–with the permission of Southern Water Ltd (U.K)).

All chlorine solutions (0.5% or 5,000 mg/L) and hydrated lime suspensions (10%, 20% and 30% w/v) were prepared in the EPHReG laboratory. Chlorine solutions were prepared from calcium hypochlorite (Ca (ClO)_2_), also known as “high test hypochlorite” (HTH 65%; Minstral^®^); sodium dichloroisocyanurate (C_3_Cl_2_N_3_NaO_3_; NaDCC or SIDC 65%; Minstral^®^); and sodium hypochlorite (NaOCl) (Bleach 1.5% Sainsbury^®^). Performance assessments were undertaken within 14 litre high-density polyethylene (HDPE) plastic buckets supplied by the international NGO OXFAM^®^. Bacterial indicators (faecal coliforms and intestinal enterococci) and viral indicators (somatic coliphages, F^+^specific bacteriophages and bacteriophages infecting *Bacteroides fragilis* (strain GB124)) were used to evaluate performance. High titre bacteriophage stocks used to “spike” the excreta matrices were provided from the EPHReG repository, and made using phages purified and concentrated by a propagation method described elsewhere [34].

### Production of excreta matrices (EM)

Human excreta consist mainly of faecal material and urine, although blood and vomitus could also be present, especially in disease outbreaks. The physical and chemical characteristics, as well as daily output of human excreta depend on age, ethnicity, disease, diet, income levels and the geographical location of the excreting population [35, 36]. Previous studies have demonstrated that a healthy person typically excretes an average of 300 grammes of faeces (wet weight) and 1,200 mL of urine per day [36, 37 and 38]. However, this ratio of faeces to urine may vary radically in excreta from patients at an ETC, since most patients are likely to produce watery, possibly bloody diarrhoea [39]. The likely concentrations of human excreta from healthy persons and from patients suffering from Ebola or cholera were subsequently used to inform the production of human excreta simulants for this study.

To produce excreta matrices for this study, raw wastewater and faecal sludge were used to simulate the liquid (urine) and solid (wet faeces) fractions, respectively. Three excreta matrices (EM), containing varying amounts of solid and dissolved organic matter, were produced. ‘EM 20%’ was composed of 80% raw wastewater plus 20% faecal sludge to represent excreta from healthy persons; ‘EM 10%’ was composed of 90% raw wastewater plus 10% faecal sludge to represent excreta from patients with mild diarrhoea; and ‘EM 0%’ was composed of 100% raw wastewater to represent excreta from patients with severe diarrhoea.

### Production of chlorine solutions and lime suspensions

First, the HACH^®^ 8209 Iodometric titration method (HACH^®^, Loveland, CO) was used in a series of tests to certify chlorine concentrations within HTH granules (65% available chlorine), NaDCC granules (65% available chlorine) and Sainsbury’s ^®^ domestic thin bleach (containing less than 5% available chlorine). Results showed different percentages to those displayed on the product labels, in that HTH and NaDCC granules recorded available chlorine levels of 58.5% and 53.7% respectively (not 65%). The bleach solution recorded an available chlorine content of 1.58%. All reagents were recently purchased, stored according to the manufacturer’s instructions and well within their use by date.

Prior to each assessment, one litre of each 0.5% standard chlorine solution was prepared as follows: One litre of fresh deionised H_2_0 was added to glass Schott bottles. Using an OHAUS^®^ Adventurer Pro digital scale (d = 0.01 g), 8.54 grammes of HTH or 9.30 grammes of NaDCC were weighed and 316.5 mL of bleach were measured. The chlorine solutions were added to the bottles and stirred using a plastic-coated stirring rod. Each solution was labelled according to the chlorine type and allowed to stand for at least 30 minutes (to achieve total compound dissociation) prior to use. A total chlorine concentration test (HACH^®^ 8209) was always performed on each chlorine solution prior to treatment assessments to certify that solutions contained 0.5% (± 0.025%) available chlorine.

The three hydrated lime (HL) suspensions were prepared as follows: ‘HL 10%’, ‘HL 20%’ and ‘HL 30%’ (w/v) by mixing a weighed quantity of lime (Rugby^®^, CEMEX U.K.) with de-ionised H_2_O in glass Schott bottles, which were used within 24 hrs.

### Experimental setup

Experiments were undertaken to simulate the human excreta handling and containment suggested by MSF, CDC and WHO [10–12 and 20] for use in ETC. Unfortunately, as mentioned, these existing protocols provide only limited information on the volume of chlorine solutions and lime suspensions required to treat per unit volume of excreta. The MSF protocol [10] provides the greatest detail on the chlorine solution volumes that should be applied to known volumes of excreta. Therefore, this information was used to calculate a standard volume of solution/suspension to be used throughout all experiments (see S1 File). The standard volume of chlorine, or lime was set as 10% of the volume of the excreta matrix, i.e., a ratio of 1:10 chlorine solution or lime suspension: excreta matrix).

**Fig 1:**
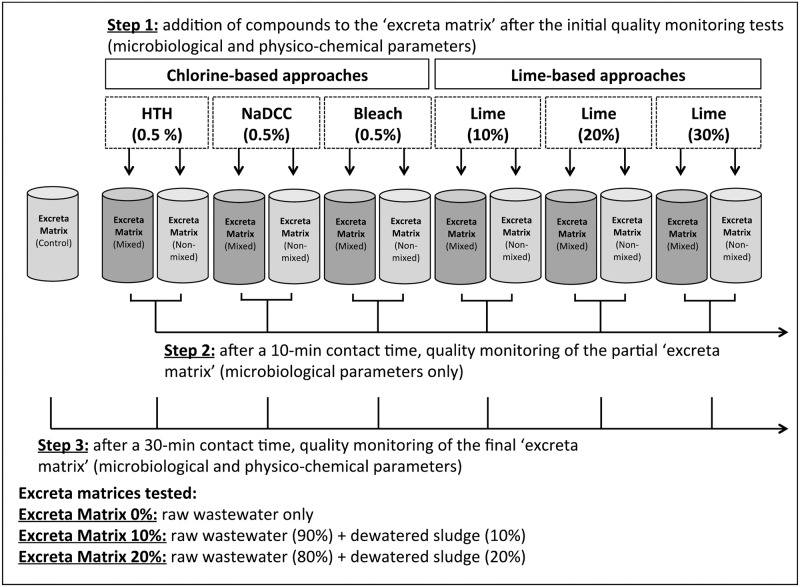
Flow chart of the human excreta containment experiments.

Personal communication from MSF personnel suggested that containment and handling of human excreta is performed within buckets filled to one third of their capacity (approximately 4–4.6 L. of excreta plus chlorine solutions or lime suspension). Proportional volumes of the previously described excreta matrices were used during the bucket experiments (EM 0% = 4,500 mL of wastewater; EM 10% = 4,050 mL of wastewater + 450 grammes of faecal sludge; and EM 20% = 3,600 mL of wastewater + 900 grammes of faecal sludge). Furthermore, 450 mLs of chlorine solution (0.5% HTH, NaDCC or bleach), or hydrated lime (HL 10%, 20% or 30%) suspensions were added to OXFAM^®^ buckets containing 4,500 grammes of excreta matrix (Fig 1). The efficacy of the three chlorine-based and three lime-based approaches was then tested using three excreta matrices (EM 0%, 10% & 20%), under mixed or non-mixed conditions and following two contact times (Ct 10 & 30 mins), as suggested by the existing WASH protocols. All tests were undertaken in triplicate for each approach (x 6), excreta matrices (x 3), mixing regimes (x 2), and contact times (x 2)(n = 216).

Faecal sludge was weighed using an OHAUS^®^ Ranger 3000 digital balance and volumes of wastewater were measured using measuring cylinders. Excreta matrices were then mixed and homogenized using an Arbeco^®^ jar test machine (with a paddle speed of 200 rpm) for approximately 3 mins and the contents distributed into the various buckets (six treatments plus one control). Because naturally occurring concentrations of F-specific and GB124 phages are relatively low (compared with the other indicators) in UK municipal wastewaters, 5 mL of pre-prepared bacteriophage stock solutions of MS2 and B124 (± 10^9^ PFU mL^-1^), respectively, were “spiked” into the buckets in order to raise the concentrations of these viral indicator to a level high enough to observe log reductions within the various excreta matrices.

All bucket studies were conducted at room temperature (approx. 21 °C). Before the addition of any chlorine solution or lime suspension, and with the intention of quantifying the initial levels of all viral and bacterial indicators, a 5 ml sample was taken randomly from the buckets and poured into 50 mL self-standing centrifuge tubes (Corning^®^), containing 45 mL of quarter strength Ringer’s (QSR) solution. For the ‘mixed’ method, the bucket contents were stirred gently for a period of ten seconds, using a plastic-coated stirring rod. Following subsequent contact times (Ct) of 10 and 30 mins, excreta matrices were subsequently and rapidly stirred for 1–2 seconds, before further 5 mL samples were withdrawn for analysis from all buckets (including the control bucket). Samples were poured into 50 ml self-standing centrifuge tubes (Corning^®^) containing either 45 mL of a sodium thiosulphate solution (300 mg/L) (BDH chemicals) for the chlorine-based treatments, or 45 ml of quarter strength Ringer’s (QSR) solution for lime-based treatments and control buckets. Dilution series (10^−2^, 10^−3^, 10^−4^) were produced from these master (10^−1^) 50 ml dilutions and analysed immediately.

In order to evaluate performance, viral and bacterial indicators were enumerated before and during treatment. Faecal coliforms (FC) and intestinal enterococci (IE) enumeration followed standard methods: namely ISO 9308/1:200035 [40] and ISO 7899/2:200036 [41], respectively. Duplicate samples were placed onto either m-fecal coliform (mFC) or m-Enterococcus (mEnt) agar (Difco^®^) in Ø 55mm Petri dishes. Results were expressed as colony-forming units (CFU) per mL. Somatic coliphages (SOMPH) were enumerated in accordance with ISO standard 10705–237 [42] and *E*. *coli* (WG5) was used as the host bacterium [43]. F-specific phages (F^+^PH) were enumerated according to ISO standard 10705–138 [44] and *Salmonella typhimurium* (WG49) [45] was used as the host bacterium. Phages infecting *Bacteroides fragilis* (GB124PH) were enumerated according to ISO standard 10705–439 [46] using strain GB-124 [47] as the host bacterium. All phage enumeration was carried out in duplicate and results were expressed as plaque-forming units (PFU) per mL. For initial and control samples, in which bacterial and viral concentrations were higher, 10^−3^, 10^−4^ and 10^−5^ dilutions were used, while for treated samples, 10^0^, 10^−1^, 10^−2^ and 10^−3^ dilutions were used. The limit of detection for all microorganisms was either 1 PFU mL^-1^ or 1 CFU mL^-1^ for EM 0%; and 10 PFU mL^-1^ or 10 CFU mL^-1^ for EM 10% and EM 20%.

### Physico-chemical analyses

Chemical oxygen demand (COD), suspended solids (TSS), ammonium ions and pH were determined in order to assess the basic characteristics of the excreta matrices. TSS and COD analyses were conducted according to APHA Standard Methods for the Examination of Water and Wastewater [48]. TSS was measured using method 2540 B and results were expressed as mg TSS.L^-1^. COD was measured using method 5220 D, and results were expressed as mg O_2_.L^-1^. The concentration of ammonium ions was measured by the indophenol blue method; analyses were performed using a HACH^®^ DR3900 bench-top spectrophotometer (HACH^®^, Loveland, USA) and a HACH^®^ LCK 303 kit (high range, 2–47 mg NH_4_^+^-N.L^-1^) according to the manufacturer’s instructions. pH levels were measured using a Mettler Toledo FE20-ATC Kit FiveEasy^™^ benchtop pH meter.

In order to certify chlorine solutions were at a concentration of 0.5% (5,000 mg L^-1^) prior to use in the full-scale bucket experiments, the HACH^®^ Total Chlorine Iodometric Method Using Sodium Thiosulphate 8209 (20 to 70,000 mg L^-1^) was used according to the manufacturer’s instructions. Miniaturised treatment experiments were performed to assess chlorine demand exerted by each excreta matrix. Excreta matrices and compound volumes were proportional and a hundred-times smaller than those used in the full-scale bucket experiments previously described. Therefore, 4.5 ml of chlorine solutions or lime suspensions were added to 50 mL centrifuge tubes containing the excreta matrices (EM 0% = 45 mL of wastewater; EM 10% = 40.5 mL. wastewater + 4.5 grammes of faecal sludge; and EM 20% = 36 mL wastewater + 9 grammes of faecal sludge). Tubes were inverted three-times to facilitate mixing. After 10 and 30 min contact times, 1 mL of sample was withdrawn and HACH^®^ method 8021 (USEPA DPD Method) was used to determine the concentration of free residual chlorine. (Note: this is a low range method (0.02 to 2.00 mg L^-1^ CL_2_) and samples were serially diluted, with 9 mL volumes of deionised H_2_O, when results were ‘over-range’. Results were expressed as mg L^-1^ CL_2_.

Assessment of pH levels of chlorine solutions, lime suspensions and treated excreta matrices were performed in parallel to the main bucket experiments using the Mettler Toledo FE20-ATC Kit FiveEasy^™^ benchtop pH meter.

### Statistical analyses

Statistical analysis of data was accomplished with the aid of the ‘IBM Statistical Package for the Social Sciences (SPSS) 23.0’, and ‘Microsoft Excel 2013’. Non-parametric statistical tests were used to analyse the main data from this research (i.e., levels of bacterial and viral indicators in all excreta matrices pre and post treatment) and median values chosen to express more accurately the average levels. Parametric statistical tests were used analyse physico-chemical data. The criterion of 95% confidence, or a 0.05 probability (p), was applied to test the significance of the various statistical tests used during this study.

The efficacy of the various approaches was evaluated by recording the final concentration of microorganisms present and calculating the log reduction of each microorganism for each approach (to the detection limit,). Additionally, calculation of log reduction was performed for the various approaches (mixed vs. non-mixed; Ct values) and also for each excreta matrix. The Kruskal-Wallis test (rank-based non-parametric one-way ANOVA) was used to determine whether there were statistically significant differences between efficacies for the various solutions and suspensions tested. Furthermore, a Dunn-Bonferroni *post hoc* paired-comparison test was performed to identify differences in efficacy between all six approaches. The Mann–Whitney two-sample rank-sum test, was used to compare differences between two independent groups of variables (namely: Ct10 vs. Ct 30 mins and mixed vs. non-mixed). ANOVA was used to determine whether there were statistically significant differences between the residual chlorine concentrations of the three chlorine solutions following addition to excreta matrices. An Independent Samples T-test was used to compare pH and residual chlorine differences between the chlorine-based and lime-based approaches.

## Results

### Overall performance

**Fig 2:**
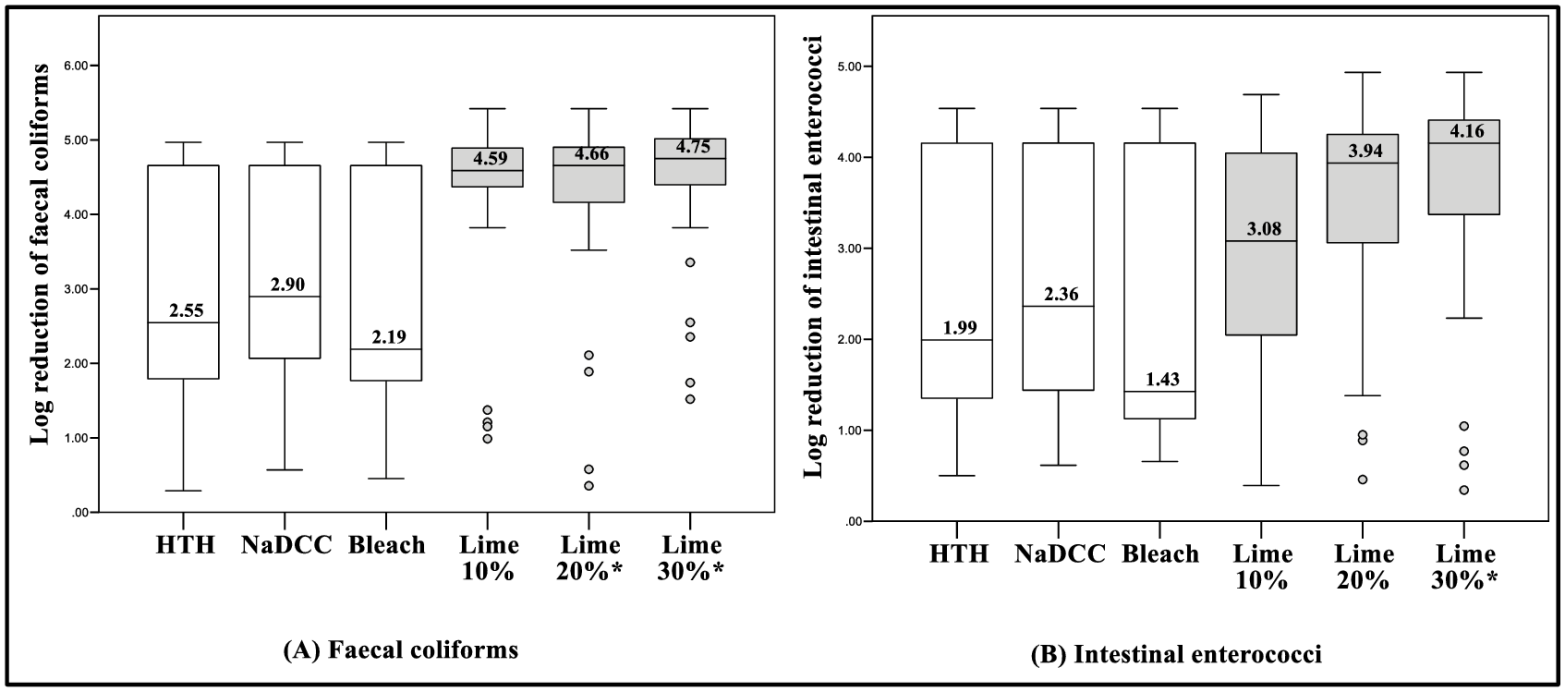
Box-plots displaying overall log reduction levels for bacterial indicators (A & B) following the addition of chlorine and hydrated lime to excreta matrices (pooled data n = 36; 2 contact times (Ct) x 2 mixing methods x 3 excreta matrices x 3 repetitions). * = Indicates a significant statistical difference in log reduction.

**Fig 3:**
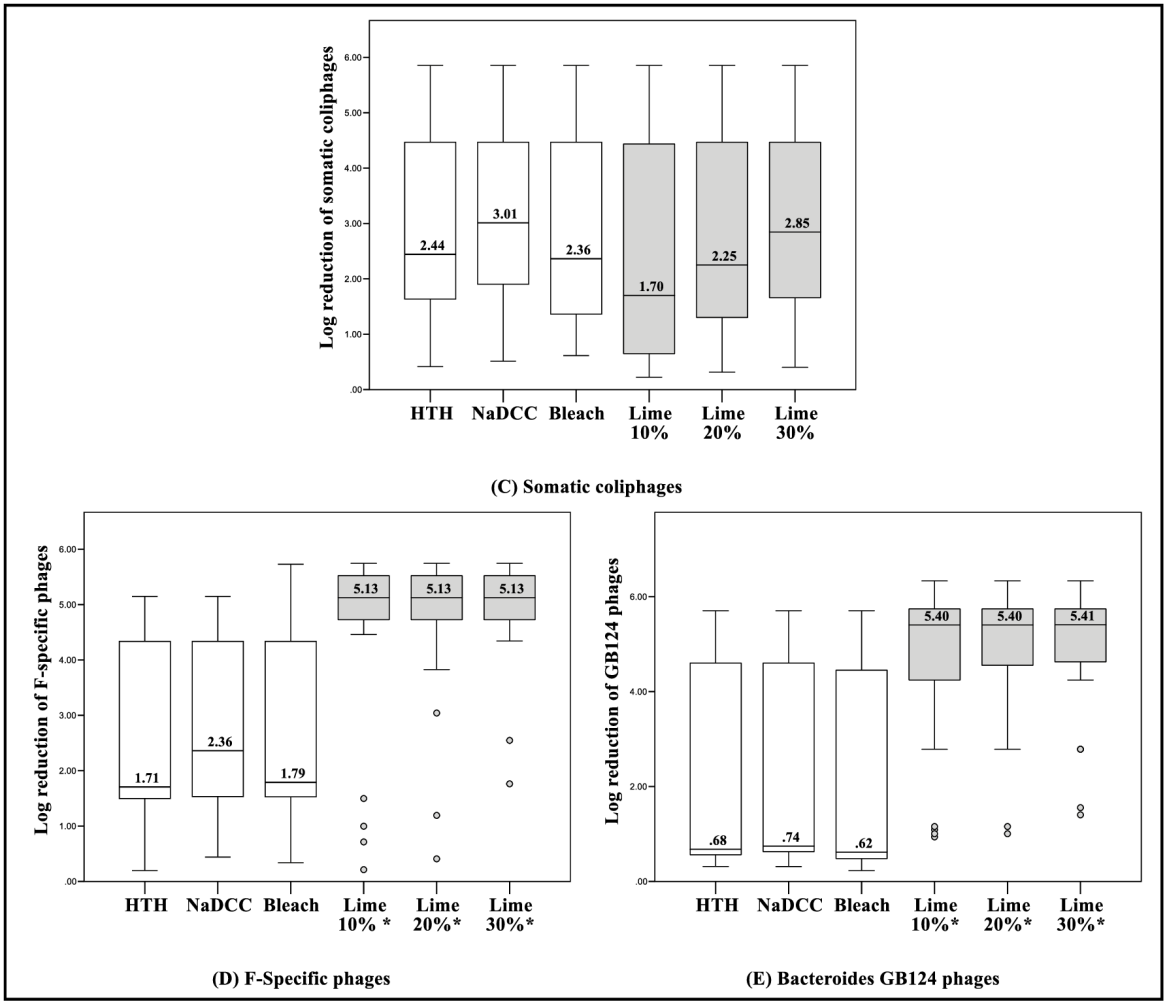
Box-plots displaying overall log reduction levels for viral indicators (C, D & E) following the addition of chlorine and hydrated lime to excreta matrices (pooled data n = 36; 2 contact times (Ct) x 2 mixing methods x 3 excreta matrices x 3 repetitions). * = Indicates a significant statistical difference in log reduction.

Overall, the average (median (range)) initial levels of indicator organisms (prior to chlorine or lime addition) were as follows: FC = 7.7 x 10^4^ (1.7 x 10^6^) CFU mL^-1^; IE = 3.5 x 10^4^ (1.1 x 10^6^) CFU mL^-1^; SOMPH = 4.9 x 10^4^ (7.1 x 10^5^) PFU mL^-1^; F+PH = 1.3 x 10^5^ (5.4 x 10^5^) PFU mL^-1^; and GB124PH = 4.6 x 10^5^ PFU mL^-1^ (2.2 x 10^6^). There was no statistical difference (p >0.05) between the average initial levels and the average final levels of microorganisms in the “control” buckets (without chlorine or lime). Average levels (median and geometric mean) of surviving microorganisms following chlorine and lime-based treatment are provided in the supporting information (S2 Table). Overall log reduction levels for each microorganism and each approach are displayed as box-plot graphs in Figs 2 and 3. These values are based on median values from pooled samples of all excreta matrices (EM 20%, 10% and 0%) and approaches, including mixed vs. non-mixed; and Ct10 vs. Ct30.

Overall, the hydrated lime suspensions demonstrated greater efficacy compared with the chlorine solutions. Statistically, for FC, HL 20% and HL30% demonstrated greater log reductions than achieved using the three chlorine-based approaches. For IE, HL 30% demonstrated greater log reduction (p<0.05) than all chlorine–based approaches.

For SOMPH, there were no significant differences in log reduction (p>0.05) between all lime- and chlorine-based approaches. For the ‘spiked’ F+PH and GB124PH, all lime suspensions demonstrated statistically greater log reduction (p<0.05) than the chlorine solutions. There was no significant statistical difference in log reduction (p>0.05) between any of the chlorine solutions (S2 File).

### Performance with respect to excreta matrix (EM)

As displayed in Figs 2 and 3—data ranges of the overall log reduction for bacterial and viral indicators were large in almost all instances. These variances are explained mainly by differences in treatments efficacy with respect to excreta matrix (Table 1). All approaches performed well against EM 0% (pure wastewater), resulting in average log reductions ranging from 4.17 to 5.29. Additionally, no significant statistical difference was found between any of the approaches against EM 0% (Table 1 and S3 File).

**Table 1:** Efficacy (median (range) log reduction) with respect to excreta matrix.

Excreta Matrix	Approach	FC	IE	SOMPH	F^+^ PH	GB124PH
**0%**	**HTH**	4.82 (.56)	4.34 (.60)	4.53 (1.53)	5.00 (.81)	5.02 (2.92)
	**NaDCC**	4.82 (.56)	4.34 (.60)	4.53 (1.67)	5.00(.81)	5.02 (2.92)
	**Bleach**	4.82 (.56)	4.34.60	4.53 (1.67)	5.07 (1.39)	5.02 (2.92)
	**Lime 10%**	4.82 (.77)	4.20 (.90)	5.03 (1.67)	5.13(1.00)	5.29(2.92)
	**Lime 20%**	4.82 (.77)	4.17(.65)	5.07 (1.67)	5.13(1.00)	5.29 (2.92)
	**Lime 30%**	4.82 (.77)	4.25(.60)	5.07 (1.67)	5.13 (1.00)	5.29 (2.92)
**10%**	**HTH**	2.36 (2.08)	1.77 (1.85)	1.83 (4.72)	1.67 (.56)	0.65 (.21)
	**NaDCC**	2.58 (1.63)	1.96 (2.01)	2.43 (3.81)	2.36 (.67)	0.71 (.15)
	**Bleach**	1.90 (1.03)	1.31 (1.06)	1.65 (4.52)	1.79 (.36)	0.48 (.25)
	**Lime 10%**	4.88* (1.60)	2.19 (3.81)	0.92 (1.67)	5.11* (1.21)	5.43* (1.98)
	**Lime 20%**	4.89* (1.90)	3.41 (3.17)	1.52 (1.63)	5.11* (1.21)	5.43* (1.98)
	**Lime 30%**	4.89* (1.60)	4.28* (2.62)	1.87 (2.05)	5.11* (1.21)	5.28* (1.98)
**20%**	**HTH**	1.53 (2.64)	1.25 (1.65)	1.98 (2.63)	1.28 (1.72)	0.56 (.51)
	**NaDCC**	2.00 (3.09)	1.36 (1.91)	1.90 (4.26)	1.48 (1.31)	0.56 (.78)
	**Bleach**	2.03 (3.17)	1.13 (1.68)	1.85 (3.54)	1.45 (1.38)	0.59 (.86)
	**Lime 10%**	4.36 (3.46)	2.91 (2.95)	1.05 (2.25)	5.29* (5.53)	5.40* (5.40)
	**Lime 20%**	4.05 (4.10)	3.64 (4.48)	1.46 (4.64)	5.29* (5.34)	5.40* (5.33)
	**Lime 30%**	4.39 (3.63)	3.71 (4.59)	1.96 (4.55)	5.29* (3.98)	5.41* (4.93)

* = Log reduction values in bold were significantly greater (p < 0.05) than other log reductions observed within that excreta matrix

Efficacy of the various approaches decreased as the organic content and suspended solids increased within the matrices (EM 10% and EM 20%). This trend was more pronounced and significant for the chlorine-based approaches.

For EM 10%, log reduction ranged from 0.48 to 2.58 for chlorine-based approaches and from 0.92 to 5.43 for lime-based approaches. Faecal coliform log reduction was statistically higher for all lime-based approaches (max. = 4.89) compared with chlorine-based approaches (max. = 2.58). For IE, HL 30% demonstrated statistically a higher log reduction (4.28) than all chlorine-based approaches (max. = 1.96) and HL 20% demonstrated a statistically higher log reduction than ‘domestic bleach’. Interestingly, the only statistical difference reported for SOMPH reduction was that the log reduction for ‘NaDCC’ (2.43) was higher than for ‘HL 10%’ (0.92). For F+PH and GB-124 phages all lime-based approaches demonstrated statistically higher log reductions (5.11; 5.28–5.43) than chlorine-based approaches (1.67–2.36; 0.48–0.71) (Table 1 and S3 File).

For the EM 20%, log reductions ranged from 0.56 to 2.03 for chlorine-based approaches and from 1.05 to 5.41 for lime-based approaches. Faecal coliform log reduction was statistically higher only when the HL 30% suspension (4.39) was compared with ‘HTH’ (1.53) and ‘NaDCC’ (2.00) chlorine solutions. For IE, the HL 20% (M = 3.64) and HL 30% (M = 3.71) suspensions demonstrated statistically higher log reductions than chlorine-based approaches (max. = 1.36), but the *post hoc* Dunn-Bonferroni pairwise comparison test did not demonstrate any adjusted significance value below 0.05. For SOMPH, no statistical difference was observed between any of the approaches and log reductions ranged from 1.05 to 1.98. For F+PH, the HL 20% (M = 5.29) and HL 30% (M = 5.29) suspensions demonstrated statistically higher log reductions than the chlorine-based approaches (max. = 1.48). For GB-124 phages, all log reductions for lime-based approaches (5.40–5.41) were also statistically higher than for chlorine-based approaches (0.56–0.59) (See Table 1 and S3 File).

### Performance with respect to contact time (Ct)

S3 Table summarizes and compares the efficacy of each chlorine and lime-based approach with respect to contact time (Ct) and with the various excreta matrices. From the 30 pairs of results presented, 19 demonstrated higher treatment efficacy with Ct 30 mins; eight demonstrated higher treatment efficacy with Ct 10 mins; and three demonstrated similar treatment efficacy for both contact times. However, there was no significant difference (p-value > 0.05) between the two Cts when the outcomes were statistically compared (see S4 File).

### Performance with respect to mixing regime

S4 Table summarises and compares the efficacy of each chlorine and lime-based approach when mixed or not with the various excreta matrices. From the 30 pairs of results displayed, 20 demonstrated higher log reductions when treatment was combined with mixing, while 10 demonstrated higher log reductions without mixing. Of the 15 chlorine-based approaches (paired results), ten demonstrated higher log reductions without mixing, and five demonstrated higher log reductions with mixing. However, when statistically compared, no significant differences were observed (p>0.05). All paired results (15) of lime-based suspensions showed greater treatment efficacy following mixing. Furthermore, these differences were statistically different for the following scenarios: log reduction of FC was statistically greater (p<0.05) for HL 20% and HL 30%; log reduction of IE was statistically greater (p<0.05) for HL 10% and HL 30%; and the log reduction of F-specific phages was statistically greater (p<0.05) for all the lime-based mixed approaches (S4 File).

### Results from physico-chemical analyses

A summary of the physico-chemical excreta matrix characteristics is presented in Table 2 below.

**Table 2:** Mean physico-chemical characteristics for the various human excreta matrices.

Physico-chemical parameters	Excreta matrix		
	0%	10%	20%
**Chemical oxygen demand (COD), mg l**^**-1**^	555 (± 61)	9,202 (±476)	16,847 (±494)
**Ammonium ions (NH**_**4**_^**+**^**), mg l**^**-1**^	61 (±6.4)	130 (±14.4)	184 (±14.1)
**Total suspended solids (TSS), mg l**^**-1**^	2,810 (±161)	9,380 (±234)	21,980 (±254)
**pH**	7.42 (±.07)	6.82 (±.04)	6.88 (±.07)

#### Chlorine demand

The concentration of chlorine solutions was consistently 0.5%, or 5,000 mg L^-1^. As solutions were diluted at a ratio of 1:10 with the excreta matrices, it was assumed that initial levels of free chlorine within the buckets would be 454.54 mg L^-1^ (5,000/11). Table 3 presents levels of residual chlorine following the effect of chlorine demand exerted by faecal excreta matrices during the contact times tested. Generally, residual chlorine concentrations were less after Ct 30 mins compared with those after Ct 10 mins (though these differences were shown not to be statistically significant (p> 0.05)). Also there was no statistical difference (p> 0.05) between the levels of residual chlorine for the various chlorine treatments (S5 File).

**Table 3:** Residual free chlorine (mg L^-1^) following the addition of chlorine solutions to excreta matrices.

Excreta matrix	Approach	Residual free chlorine (mg l^-1^ cl_2_)	
		Ct 10 mins	Ct 30 mins
**0%**	HTH	213.5 (± 90.9)	187.5 (± 76.1)
	NaDCC	241.3 (± 85.9)	209.6 (± 77.1)
	Bleach	208.6 (± 86.9)	204.8 (± 94.2)
**10%**	HTH	2.31 (± 0.49)	1.11 (± 0.77)
	NaDCC	2.01 (± 1.00)	1.61 (± 0.42)
	Bleach	2.06 (± 0.81)	1.68 (± 0.53)
**20%**	HTH	0.98 (± 0.75)	0.83 (± 0.50)
	NaDCC	0.86 (± 0.43)	0.88 (± 0.47)
	Bleach	0.76 (± 0.53)	0.80 (± 0.55)

It is clear that as the levels of organic matter and suspended solids increase in the excreta matrix, the higher becomes the chlorine demand exerted. Initial levels of free chlorine declined from 454.54 mg L^-1^ to residual free chlorine levels ranging from 187.5 to 241.3 mg L^-1^ in EM 0%; 1.11 to 2.31 mg L^-1^ in EM 10% and 0.76 to 0.98 mg L^-1^ in EM 20%.

#### pH

The chlorine solutions and lime suspensions exhibited mean pH values of 7.90 (HTH); 6.17 (NADCC); 11.81 (bleach); 12.90 (HL 10%); 12.91 (HL 20%) and 12.93 (HL 30%). Mean pH values of excreta-treatment mixes did not vary significantly with respect to contact time (p> 0.05). There were a few statistically significant differences between pH values of mixed and non-mixed tests (p< 0.05). Mean pH levels of excreta-treatment mixes according to excreta matrix, treatment and mixing method are presented in Table 4 below.

**Table 4:** pH values of excreta-treatment mixes according to mixing regime.

Excreta matrix	Approach	pH	
		Mixed	Non-mixed
**0%**	HTH	6.19 (± 0.23)	5.87 (± 0.06)
	NaDCC	5.91 (± 0.11)	5.94 (± 0.16)
	Bleach	6.69 (± 1.02)*	7.80 (± 0.26)*
	HL 10%	12.92 (± 0.32)	12.73 (± 0.16)
	HL 20%	12.98 (± 0.31)	12.87 (± 0.29)
	HL 30%	12.99 (± 0.30)	12.90 (± 0.06)
**10%**	HTH	6.15 (± 0.48)	6.47 (± 0.48)
	NaDCC	5.84 (± 0.17)	5.93 (± 0.18)
	Bleach	6.31 (± 0.93)*	6.70 (± 0.38)*
	HL 10%	12.33 (± 0.39)	12.26 (± 0.24)
	HL 20%	12.63 (± 0.23)	12.63 (± 0.16)
	HL 30%	12.79 (± 0.12)	12.79 (± 0.59)
**20%**	HTH	6.36 (± 0.64)	6.19 (± 0.69)
	NaDCC	6.30 (± 0.51)	6.21 (± 0.59)
	Bleach	6.82 (± 0.63)	6.81 (± 0.47)
	HL 10%	12.67 (± 0.21)	12.22 (± 0.36)
	HL 20%	13.02 (± 0.19)**	12.58 (± 0.06)**
	HL 30%	12.99 (± 0.11)	12.83 (± 0.12)

* = Denotes a significant difference (p < 0.05) in values obtained between chlorine-based approaches within that excreta matrix;

** = Denotes a significant difference (p < 0.05) in values obtained between lime-based approaches within that excreta matrix

Generally, pH values for the lime-excreta combinations were always higher when mixing was employed, although this assumption was only statistically supported for the EM 20%—HL 20% combination (S6 File).

## Discussion

This laboratory-based study aimed to assess the performance and suitability of on-site approaches for the safe handling and containment of human excreta using current WASH guidelines recommended for use in emergency settings. In addition to elucidating the suitability of existing chlorine-based approaches [10–12] (chlorination at 0.5%), the study also aimed to explore the suitability of a recently proposed lime-based excreta containment approach [20] (involving 10% lime suspensions).

The findings revealed significant differences between the approaches tested, with the results demonstrating that hydrated lime suspensions achieved the greater efficacy in containing the human excreta matrices, compared with the various chlorine solutions. Overall, the HL 10% displayed greater log reductions than the approaches involving 0.5% chlorine solutions for four of the five microbiological indicators tested (Figs 2 and 3). HL 10% provided greater log reduction of FC (+1.69), IE (+1.09), F+PH (+2.77) and GB124PH (+4.66), but a lower log reduction of SOMPH (-1.31), compared with the chlorine-based approaches. Furthermore, the difference in log reduction of F+PH and GB124PH between chlorine and HL 10% was statistically significant.

Because WASH field recomendations can vary markedly from standard operating practices in emergency settings, it was decided also to evaluate the impact of higher concentrations of hydrated lime (HL 20% and HL 30%) upon performance. These higher concentrations demonstrated higher log reductions, especially HL 30% (overall log reductions of FC = 4.75, IE = 4.16, SOMPH = 2.85, F+PH = 5.13 and GB124PH = 5.4 (Fig 2)); which showed a greater log reduction than the chlorine-based approaches for FC (+1.85), IE (+1.8), F+PH (+2.77) and GB124PH (+4.61). Again, log reductions for four of the microbiological indicator organisms were statistically higher. The HL 30% log reduction for SOMPH was also higher, being only 0.16 log lower than the best performing chlorine-based approach (overall log reductions were FC = 4.75, IE = 4.16, SOMPH = 2.85, F+PH = 5.13 and GB124PH = 5.40). Therefore, highly caustic concentrated hydrated lime suspensions appear to create a protective layer capable of containing pathogens within human excreta matrices.

There was no statistical difference in terms of performance between any of the chlorine-based approaches (HTH, NaDCC and bleach). Their highest log reduction levels were for NaDCC, were as follow: FC = 2.90, IE = 2.36, SOMPH = 3.01, F+PH = 2.36 and GB124PH = 0.74 (Figs 2 and 3). This is an important finding, which demonstrates that as long as chlorine solutions contain 0.5% available free chlorine, their performance in terms of the safe handling of excreta is comparable. However, the findings of this study also demonstrated that the concentration of available chlorine after preparing the solutions appeared to be substantially lower than those listed on the product labels (NADCC, value listed = 65%, value available = 58.5%; HTH, value listed = 65% value available = 53.7%; Bleach, value listed < 5%, value available = 1.58%). This should serve as a reminder to healthcare practitioners using chlorine-based products in the field, not to assume that the chlorine concentrations listed by the supplier are completely accurate and that the figures quoted are likely to be considerably lower than those stated. Where possible, the accuracy should be checked using a range of commercially available methods for testing chlorine solution concentrations in emergency settings [49]. Furthermore, the shelf-life of chlorine solutions recommended for cholera and Ebola disease treatment centres should also be taken into consideration by healthcare practitioners, as chlorine concentrations tend to reduce with time [50].

Chlorine-based approaches reduced the levels of microorganisms contained in the supernatant layer in the buckets, but only performed well in the excreta EM 0% (raw wastewater), their performance being significantly lower than the various lime-based approaches in the EM 10% and 20% (Table 1). This was to be expected as chlorine-based disinfectants have been previously shown to lose their bactericidal and virucidal properties rapidly when in contact with high levels of organic matter [16]. During this study, the excreta matrices exerted a significant chlorine demand and residual chlorine concentrations after 30 mins contact time ranged from 1.11 to 1.68 mg L^-1^ (EM 10%) and from 0.8 to 0.83 mg L^-1^ (EM 20%) (Table 3). It is also recognised that the attachment of microorganisms to surfaces promotes their survival in chlorinated waters [51] and the high levels of suspended solids in EM 10% and 20% (see Table 2) may also partly account for the decrease in performance observed. This seems also to apply to the lime-based approaches, although less noticeably. Here, solids may act as a shield, protecting the microorganisms from the potentially lethal alkaline environment created by the lime suspensions.

WASH treatment protocols [10–12 and 20] suggest contact times of 10, 15 and 30 mins for human excreta. Our testing of contact times (Ct 10 and 30 mins) suggested that after 10 mins, most of the removal and inactivation is likely to have already occurred (though an extended contact time of 30 mins might achieve further indicator (and pathogen) reduction, which may have been masked by the detection limits of the indicators monitored).

Given the increased risks to healthcare workers associated with any potential disturbance of buckets contents, the observation that mixing had no statistically significant effect on the efficacy of chlorine-based approaches is useful to note. Mixing did appear to slightly improve the performance of the lime-based approaches (S4 Table), which may be explained by the fact that, generally, pH levels were slightly higher in the mixed lime treatments (Table 3) and that the process of mixing is likely to increase the likelihood of the indicator organisms coming into contact with the lime compound. From the perspective of operator safety (and others in close proximity), spillage during the mixing/handling stage may actually negate any improvements in handling safety offered by mixing the bucket contents. However, results from this study suggest that the mixing of hydrated lime and human excreta could be an option for emerging sanitation treatment technologies in emergency settings [52].

Of the bacterial indicators assessed in this study, IE appeared to be more resistant to chlorine and lime-based approaches than FC under all conditions, a finding that agrees with those of previous studies [53]. Of the viral indicators, the ‘spiked’ F^+^PH and GB124PH showed considerable resistance to chlorination, also in accordance with the results of previous studies [53,54]. SOMPH demonstrated higher resistance to lime compared with all other microorganisms used in this study. Somatic coliphages are a heterogeneous group with members belonging to the families *Myoviridae*, *Siphoviridae*, *Podoviridae* and *Microviridae*. In this study, the authors noted that the lime and chlorine tolerant SOMPH phages were all similar in terms of their plaque size and shape, possibly suggesting that they may belong to the same virus family. However, further analysis of the morphology and classification of these resistant phages using electron microscopy was considered beyond the remit of this study.

According to the extant literature, the observed differences in resistance among the various bacterial and viral indicators can be attributed to their mechanisms of removal and inactivation. For instance, the main mechanisms of bacterial disinfection by chlorine and lime-based treatments are chemical reactions that lead to the lysing of microorganism cell walls. OH^-^ ions present in lime suspensions saponify lipids in the enveloping membrane [55], while the electronegativity of chlorine solutions has been shown to oxidise and denature bacterial cell wall proteins [56]. In contrast, for viruses (including phages) it is more difficult to elucidate the precise mechanism of removal and inactivation, but both chlorine and lime based approaches appear to inactivate viruses by reacting with viral proteins and genomic material [57, 58].

However, whilst studies have shown that the Ebola virus is highly sensitive to chlorine-based approaches, demonstrating a 6.6 log reduction when exposed to 0.5% chlorine on surfaces [14], its behaviour and resistance is less well understood in more complex excreta matrices, especially where organic loading may be high. *Pseudomonas syringae* phage Phi6 has been proposed as a potential surrogate for the Ebola virus in a recent study [59], as it has demonstrated a greater log reduction than the F+ specific phage MS2 (M = 4.1 and 3.2, respectively). However, given that the recent West Africa Ebola outbreak demonstrated that the WASH sector needs to be far better prepared for all future infectious disease outbreaks of as yet unknown aetiology, the authors believe that although phage Phi6 may indeed provide useful information [60], adopting a more conservative indicator(s) such as SOMPH and F^+^PH for assessing removal is an essential precautionary measure.

Research has also shown that laboratory-grown microorganisms may be more susceptible to treatment processes than indigenous microorganisms [61, 62]. This study therefore focused on a combination of both indigenous (FC, IE, SOMPH, GB124PH and F+PH) and artificially-seeded organisms (MS2 (F+PH) and GB124PH). Another potential influence on treatment efficacy is temperature, which has been shown to affect the removal and or inactivation process kinetics of certain microbes [63], possibly as a result of viral capsid damage and increased production of harmful metabolic by-products [64]. Therefore, it may be useful for future studies to investigate the effects of temperatures above typical laboratory room temperature (which was approx. 21 °C here). This may better represent the temperatures experienced in emergency situations in many arid and tropical climates. Though it is possible the final log removal values achieved may be slightly higher in such instances, it should be noted that the primary aim of this study was to compare the performance and suitability of chlorine and lime-based approaches for the safe handling and containment of human excreta in low resource settings.

This research has demonstrated that although chlorination (at 0.5% concentration) using a range of chlorine-based products appears to be effective when handling raw wastewater, these products are considerably less effective at ensuring the safe handling, containment and removal of more concentrated forms of human excreta (containing greater concentrations of organic matter). Furthermore, chlorination of human excreta also raises other safety concerns, such as the potential re-growth of resistant pathogenic microorganisms [65]. However, chlorine remains a very important and widely-used disinfectant, which clearly will continue to have a pivotal role to play in the treatment of drinking water and the cleaning of surfaces during infectious disease outbreaks. Therefore, it is suggested that effective emergency WASH response protocols should include the provision of both traditional chlorine-based approaches as well as those involving suspensions of hydrated lime, if the risks of onward transmission of infectious disease is to be minimised effectively in such situations.

However, production of lime suspensions requires larger volumes of hydrated lime compared with the equivalent volume of chlorine compounds required to produce the solutions used in this study. Transportation of large quantities of hydrated lime powder may therefore be considered a logistical problem in emergency settings. Nevertheless, lime powder is a very common building material, which is available in most parts of the world. Preparing and handling lime suspensions is relatively easy, but, like chlorine-based approaches requires the use of basic PPE. Furthermore, lime suspensions appear to demonstrate a relatively long shelf-life and maintain their high pH levels for weeks. The disposal of highly caustic hydrated lime wastes may of course cause an adverse effect on the environment. Therefore, hydrated lime sludge should be disposed in landfills sites or waste stabilization ponds. However, hydrated lime is commonly used as a soil conditioner where soil pH is low and is extensively used to raise soil pH in agricultural settings. Consequently, these effects are unlikely to be too damaging to the environment in the longer term.

Supporting our study, recent research [33] has highlighted lime to be a preferred emergency sanitation treatment, citing operational advantages that include: low cost, operational stability under temperature variation and aerobic conditions and short sanitation time. Therefore, the international WASH sector would be wrong to disregard the use of hydrated lime for the safe handling and containment of human excreta. We recommend its inclusion in emergency WASH response protocols for dealing safely with human excreta and in inventory lists for emergency settings.

Several groups are known to be currently researching alternative approaches for the safe handling, containment, disposal and treatment of human excreta. However, the results from this study are very encouraging, but further research is still required (e.g.: testing stronger chlorine concentrations; to find approaches that might be active against an even greater range of enteric microorganisms (and their surrogates, e.g., coliphages) without constituting a public health hazard; and to establish optimum ratios and contact times between solutions/suspensions and excreta matrices). Therefore, it is important that the international scientific community continues to explore alternative options with which to develop a ‘toolbox’ of methods to ensure that risks to human health are minimized across all potential disease transmission pathways within emergency settings.

## Supporting information

S1 File Rationale for the calculation of chlorine volume used in the bucket experiments. (DOCX)

S2 File Kruskal-Wallis statistical tests for overall approaches efficacy. (DOCX)

S3 File Kruskal-Wallis statistical tests for approaches efficacy according to excreta matrices. (DOCX)

S4 File Man-Witney statistical tests comparing approaches efficacy according to contact time (Ct) and mixing treatment. (DOCX)

S5 File Statistics tests related to chlorine demand. (DOCX)

S6 File Statistics tests related to pH. (DOCX)

S7 File Raw data. (XLSX)

S1 Table Summary of the recommendations for safe handling, containment and removal of human excreta in Ebola Treatment Centres. (DOCX)

S2 Table Average levels (median and geometric mean) of surviving indicator organisms according to each approach. (DOCX)

S3 Table Approaches efficacy (median log reduction) according to contact time. (DOCX)

S4 Table Approaches efficacy (median log reduction) according to mixing regime. (DOCX)
