# Supplementary material for: Assessment of recommended approaches for containment and safe handling of human excreta in emergency settings
Source: PLoS One. 2018 Jul 26;13(7):e0201344. doi: 10.1371/journal.pone.0201344 (PMC6062132; doi:10.1371/journal.pone.0201344)
Supplement: S1 File — (DOCX) [file pone.0201344.s001.docx]

# S1 File. Rationale for the calculation of chlorine volume used in the bucket experiments

**Recommendation:**

“Collect waste in a bucket with 2 cm of 0.5 % chlorine solution. Add 0.5 % chlorine with a cup sufficient to cover the waste in a bucket for 15 minutes".

From this MSF recommendation above, and assuming that the buckets used in ETC have a radius of 11 cm (as is the case for ‘OXFAM buckets’), it can be concluded that at least 759.88 ml of chlorine solution is used for containment of human excreta (see formula below).

Volume of chlorine solution per bucket = π * r^2^ * h

π = 3.14; r = 11 cm; and h = 2 cm

Volume of chlorine solution per bucket = 3.14 * 121 cm^2^ * 2 cm

Volume of chlorine solution per bucket = 759.88 cm^3^ or 760 ml.

Instalment

Personal communication with NGO WASH operatives has suggested that the majority of excreta from ETC and CTC are liquid or semi-liquid in nature. Therefore, they require only a few millilitres of chlorine to cover them. In order to create a standard volume of chlorine solution for experimental analyses, 140 ml of chlorine solution were added to the excreta matrix, in addition to the 760 ml of chlorine solution initially added to the bucket (equivalent to ‘2 cm’), totalling 900 ml. It was assumed that each bucket is used to contain and remove 9 litres of excreta. Therefore, the chlorine solution used in the field is estimated to correspond to 10 % of the excreta volume.
