# Supplementary material for: Assessment of recommended approaches for containment and safe handling of human excreta in emergency settings
Source: PLoS One. 2018 Jul 26;13(7):e0201344. doi: 10.1371/journal.pone.0201344 (PMC6062132; doi:10.1371/journal.pone.0201344)

**S2 File. Kruskal-Wallis statistical tests for overall approaches efficacy**

| **Hypothesis Test Summary** | | | | |
| --- | --- | --- | --- | --- |
|  | **Null Hypothesis** | **Test** | **Sig.** | **Decision** |
| **1** | The distribution of Log red. FC is the same across categories of Disinfectant. | Independent-Samples Kruskal-Wallis Test | .000 | Reject the null hypothesis. |
| **2** | The distribution of Log red. IE is the same across categories of Disinfectant. | Independent-Samples Kruskal-Wallis Test | .000 | Reject the null hypothesis. |
| **3** | The distribution of Log red. SOMPH is the same across categories of Disinfectant. | Independent-Samples Kruskal-Wallis Test | .363 | Retain the null hypothesis. |
| **4** | The distribution of Log red. F+PH is the same across categories of Disinfectant. | Independent-Samples Kruskal-Wallis Test | .000 | Reject the null hypothesis. |
| **5** | The distribution of Log red. GB124PH is the same across categories of Disinfectant. | Independent-Samples Kruskal-Wallis Test | .000 | Reject the null hypothesis. |
| Asymptotic significances are displayed. The significance level is .05. | | | | |


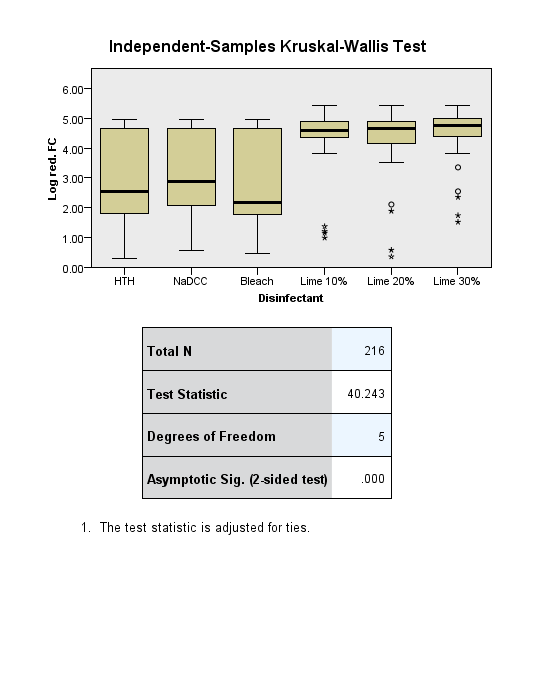


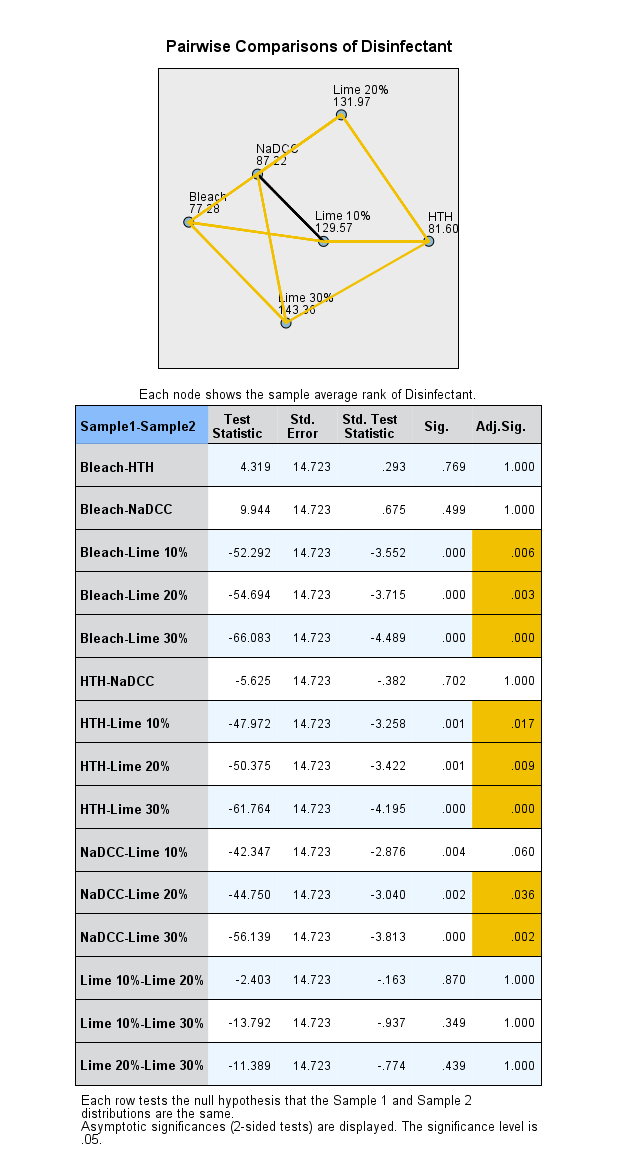


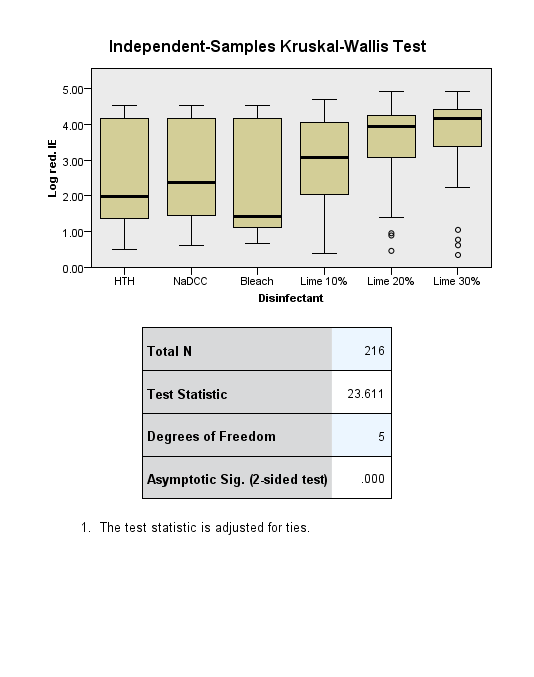

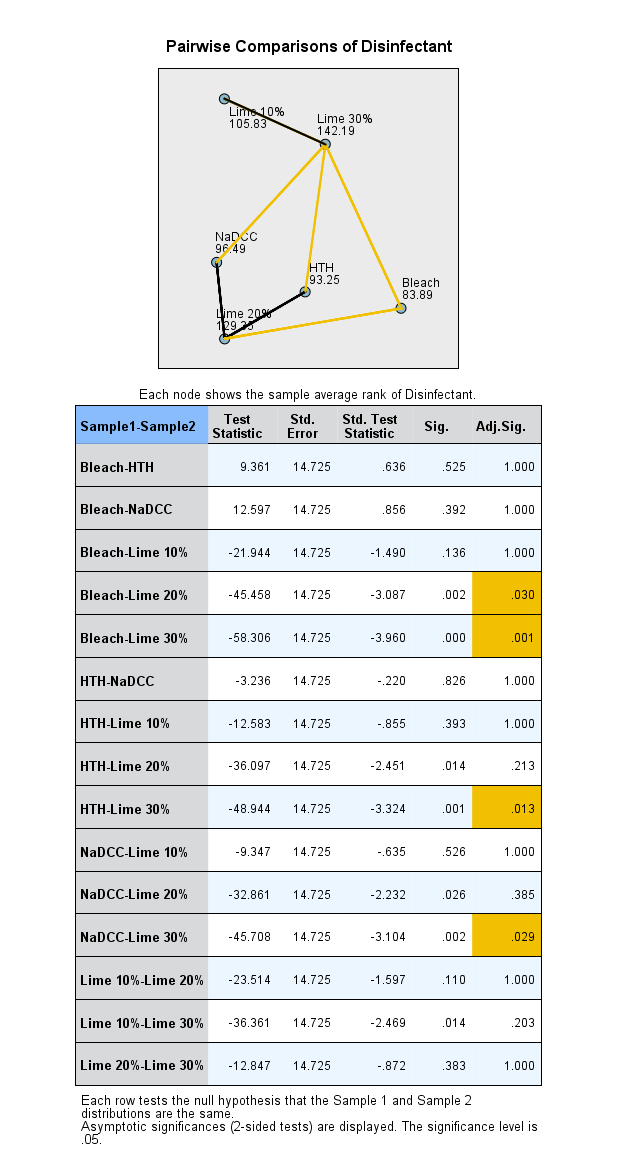


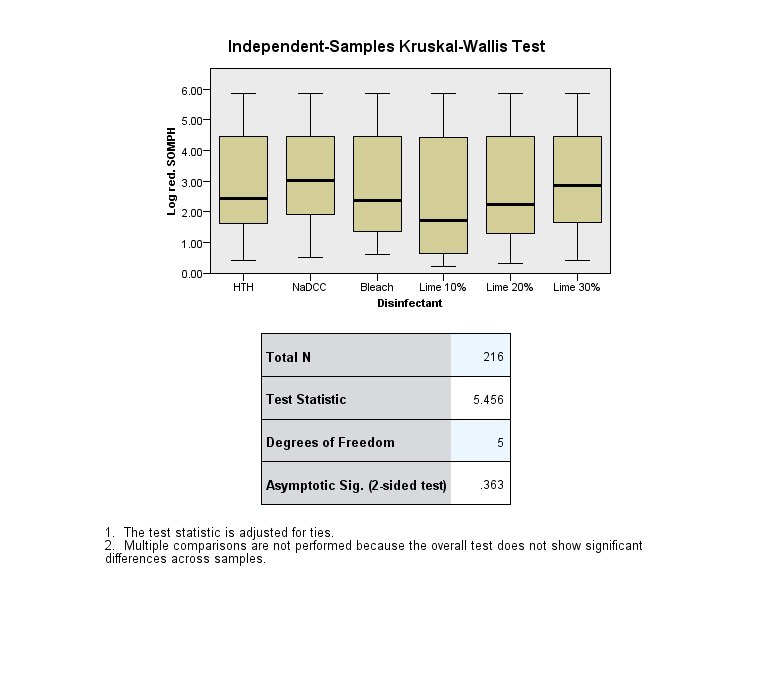

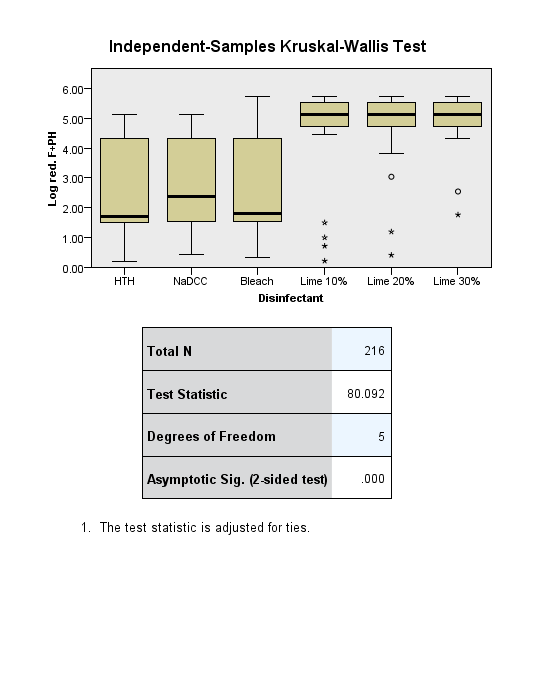

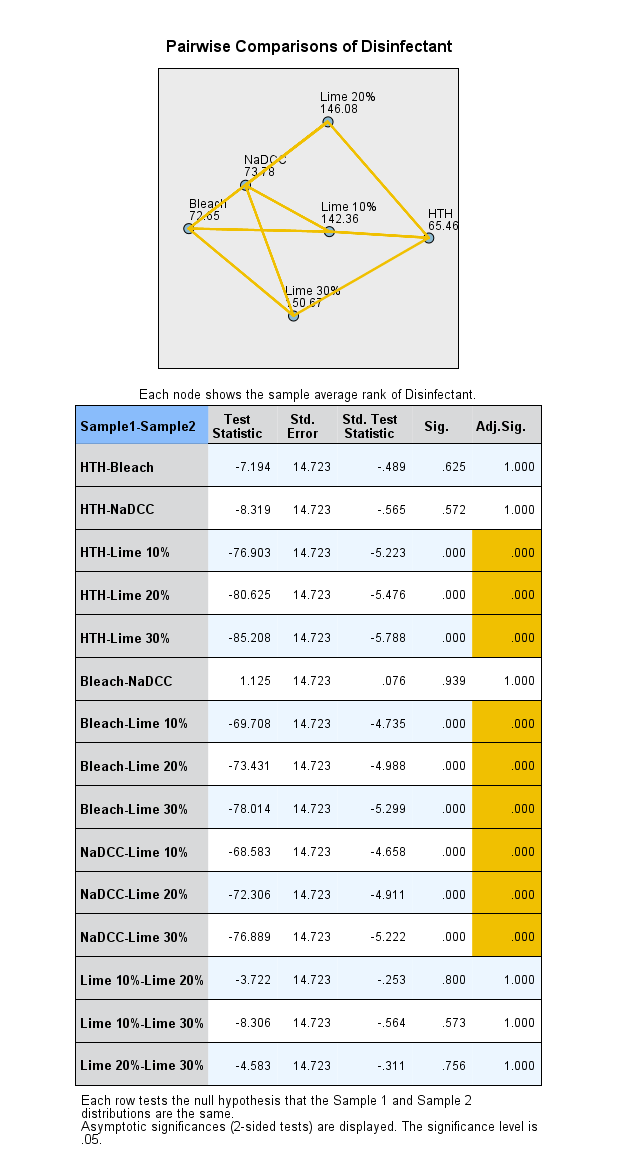


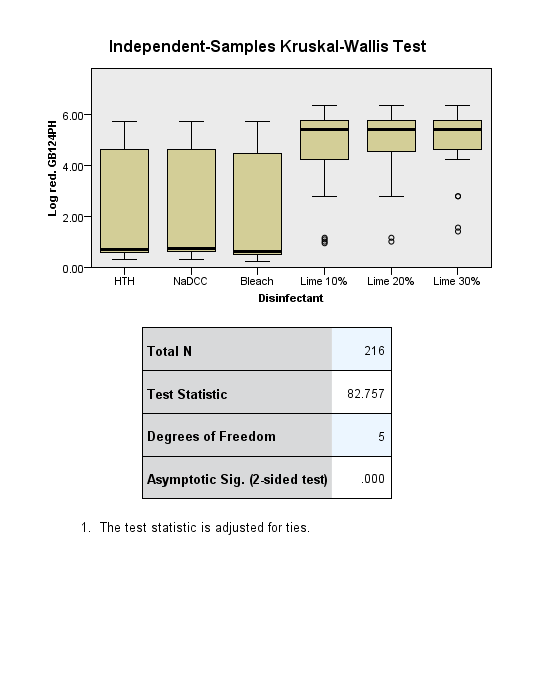


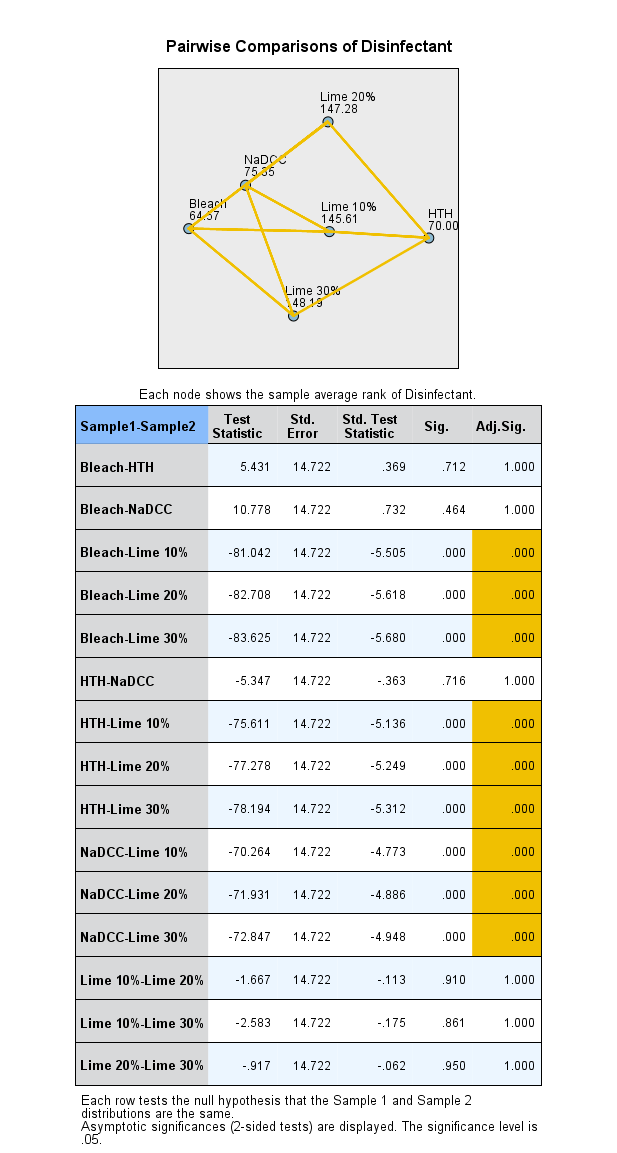

Supplement: S2 File — (DOCX) [file pone.0201344.s002.docx]
