# Supplementary material for: Assessment of recommended approaches for containment and safe handling of human excreta in emergency settings
Source: PLoS One. 2018 Jul 26;13(7):e0201344. doi: 10.1371/journal.pone.0201344 (PMC6062132; doi:10.1371/journal.pone.0201344)
Supplement: S3 File — (DOCX) [file pone.0201344.s003.docx]

**S3 File. Kruskal-Wallis statistical tests for approaches efficacy according to excreta matrices**

**Matrix 0%**

**Matrix 10%**

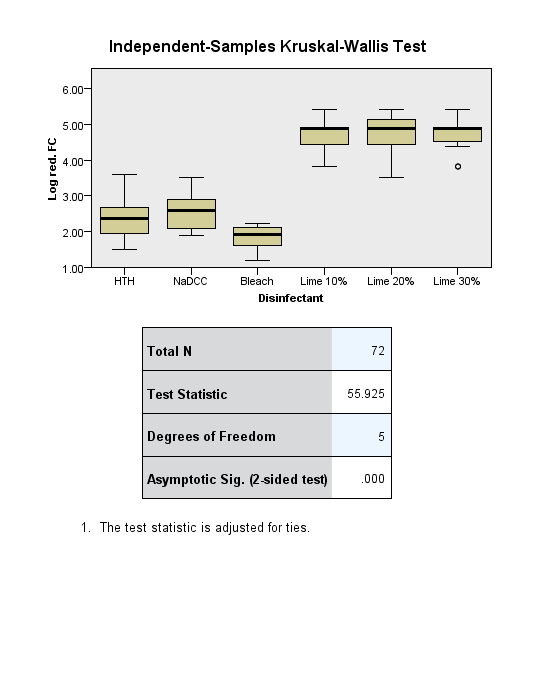


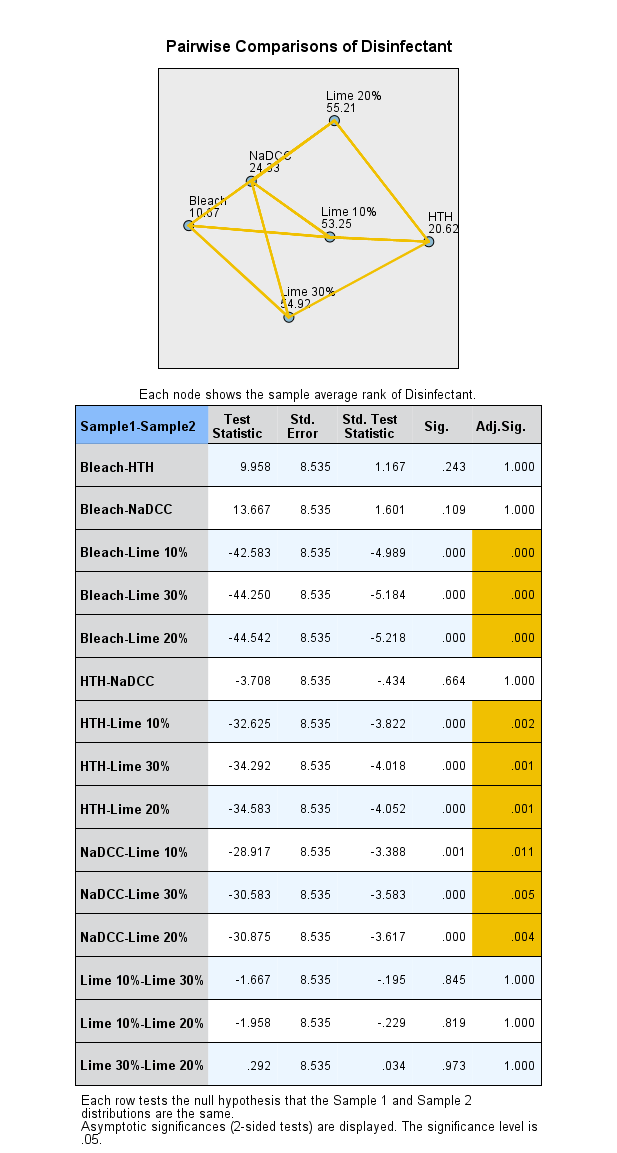


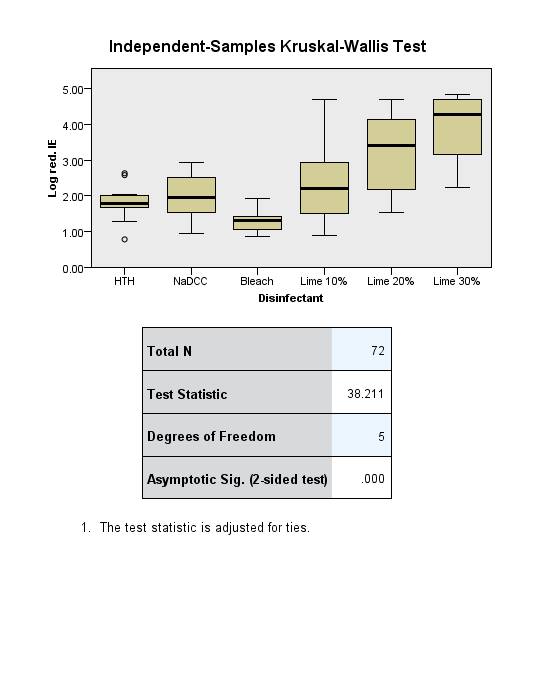


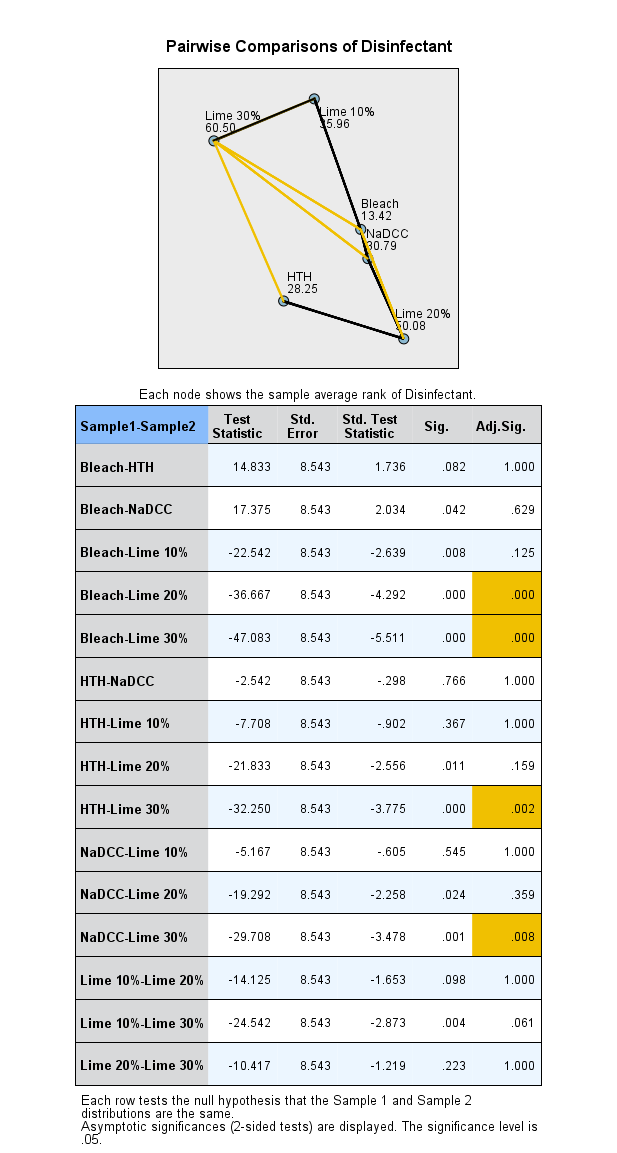


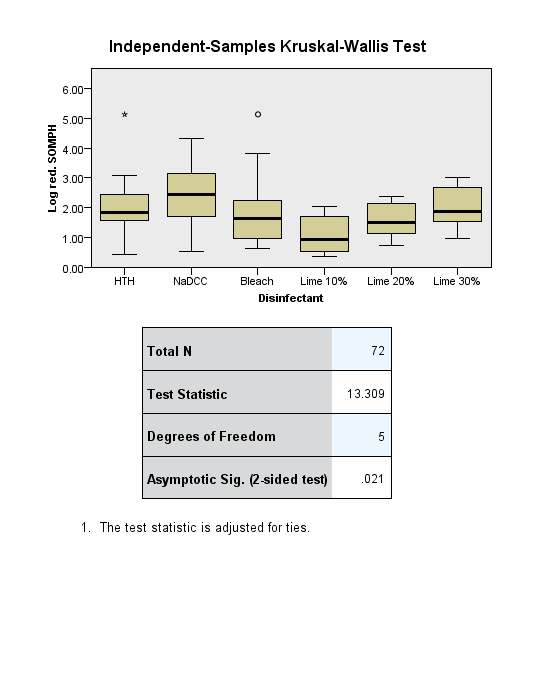


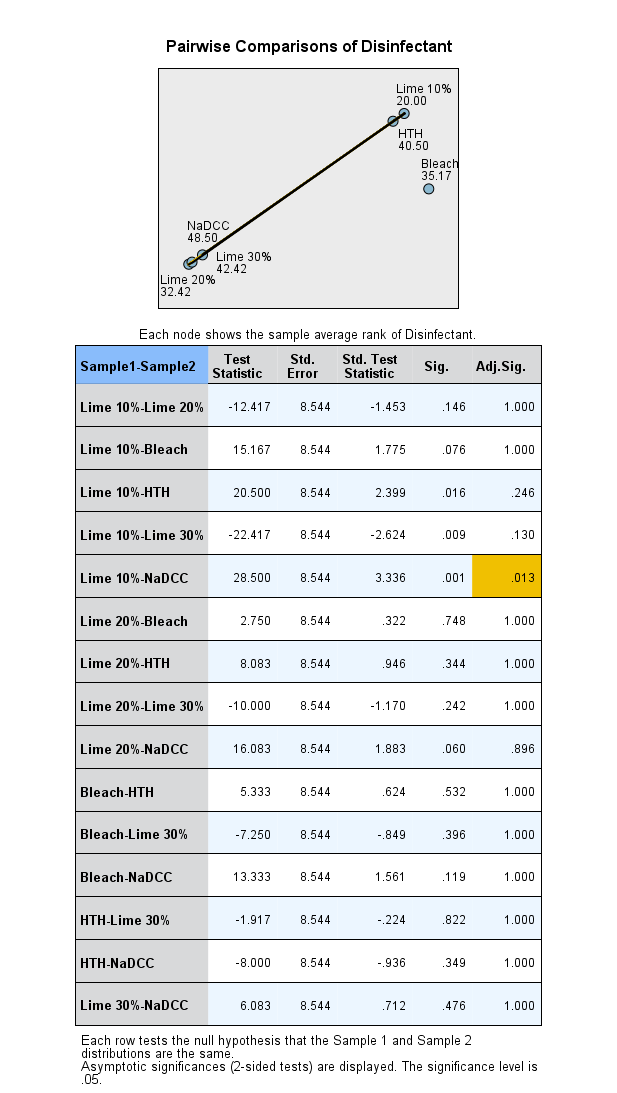


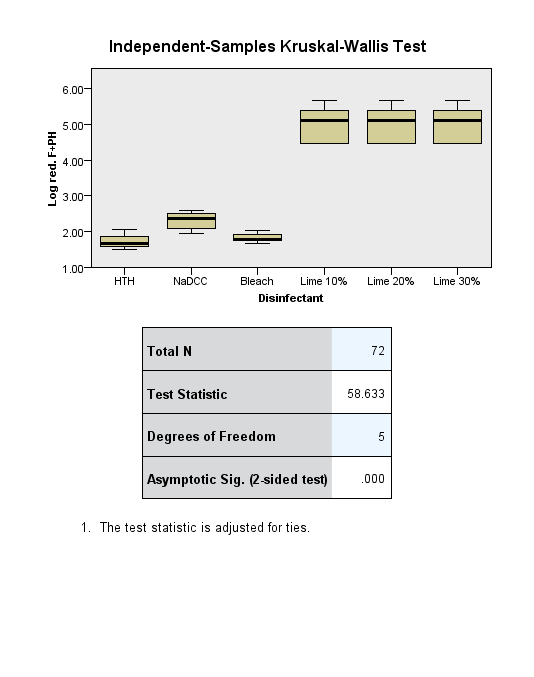


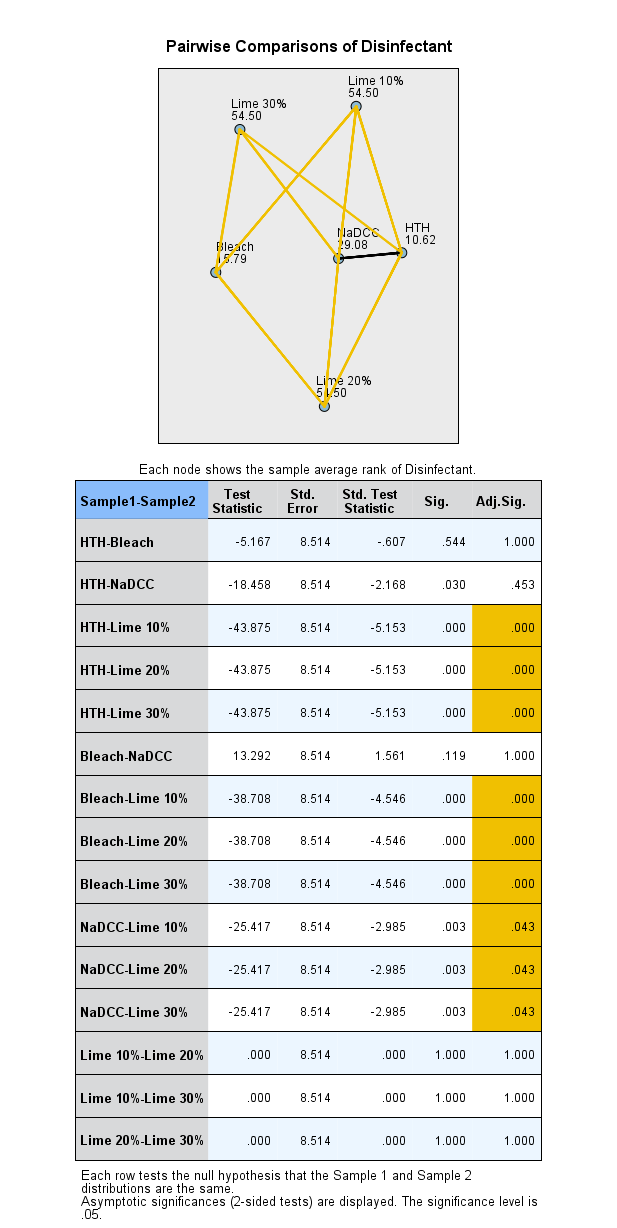


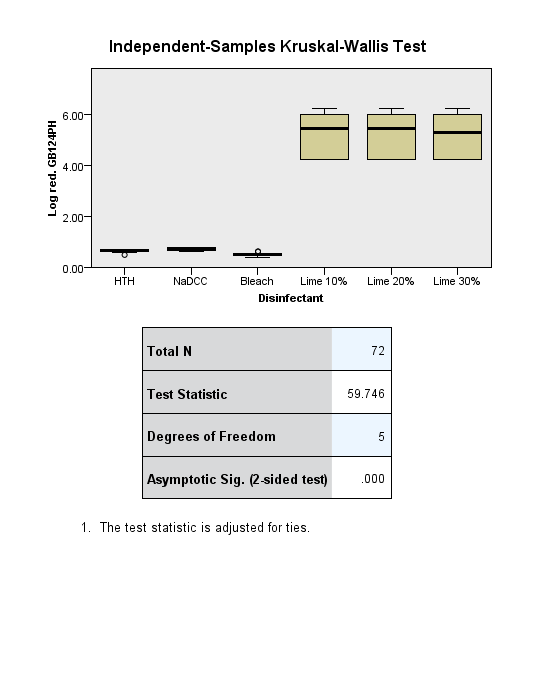


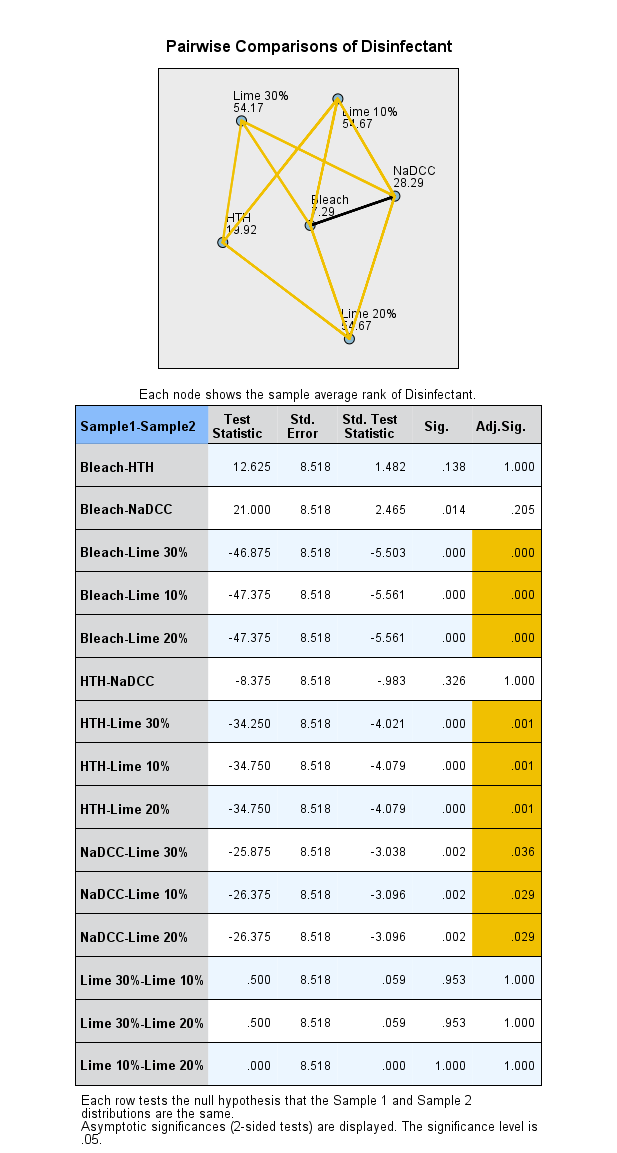


**Matrix 20%**

**
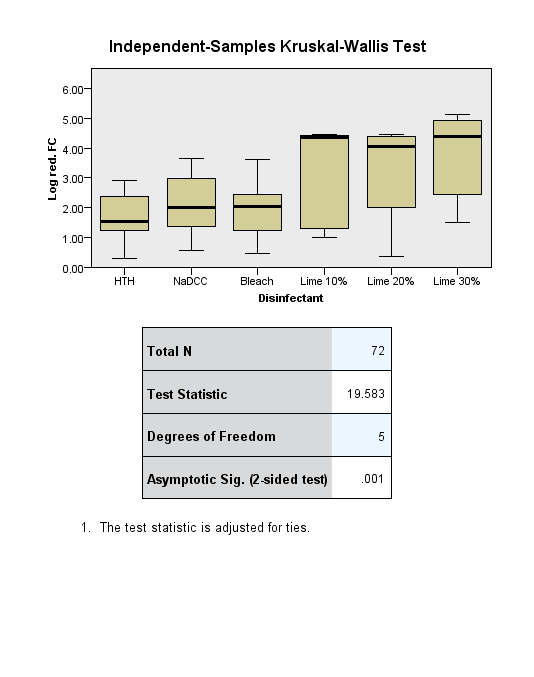
**

**
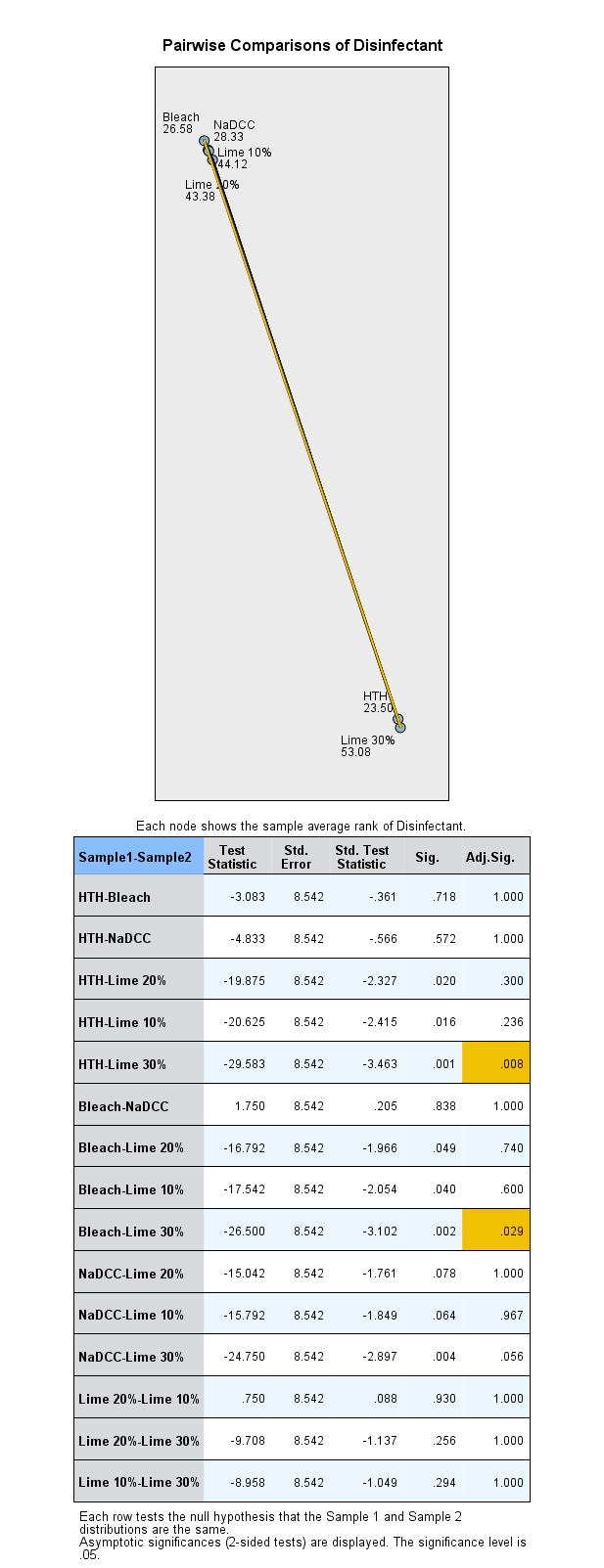
**

**
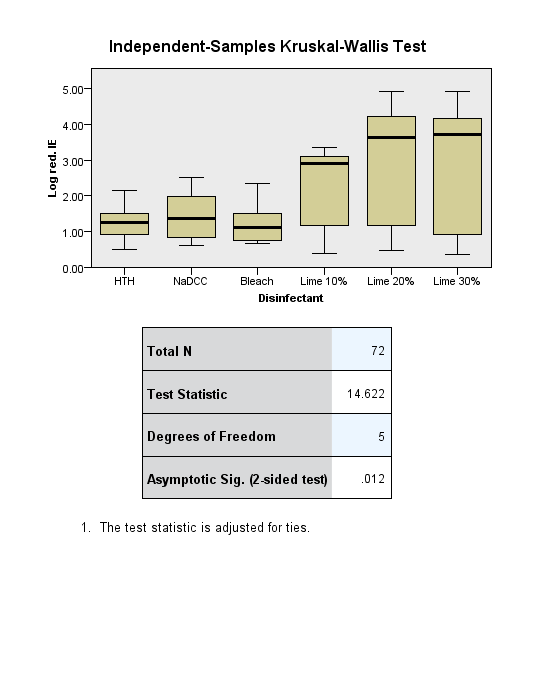
**

**
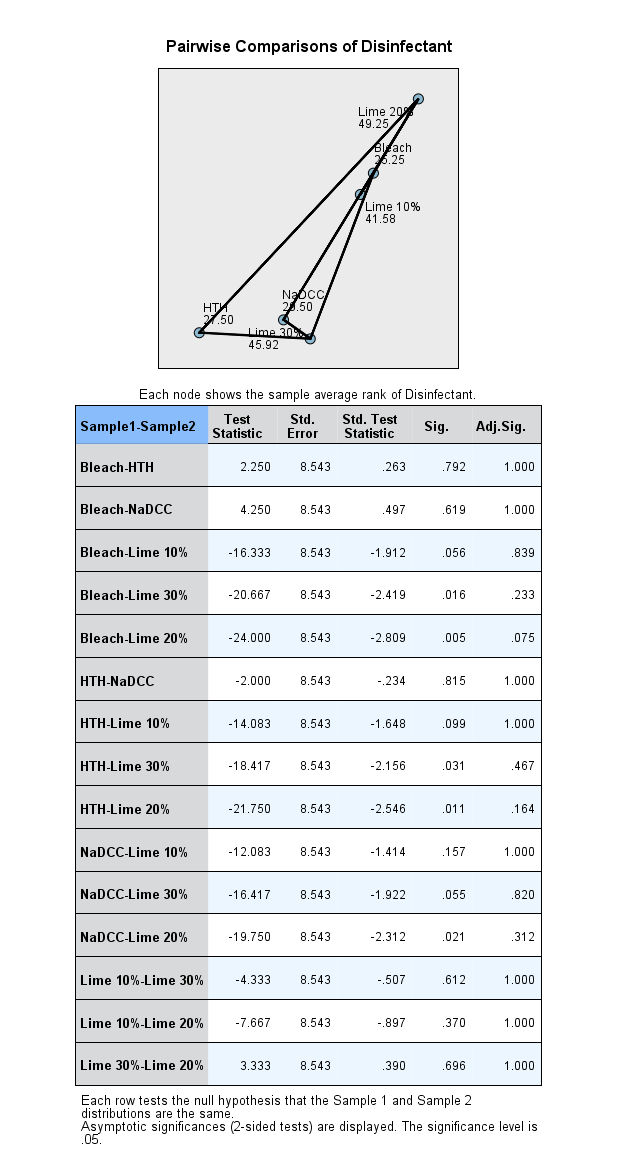
**

**
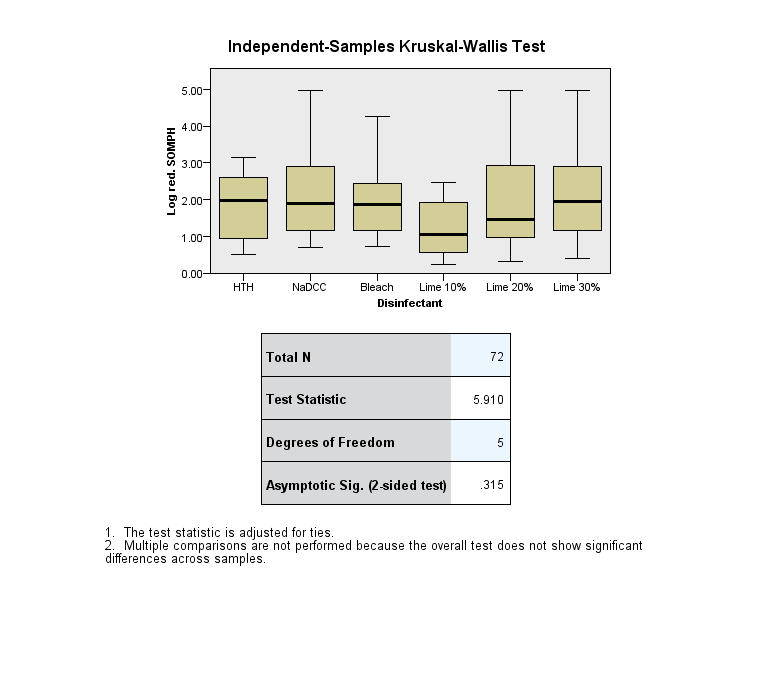
**

**
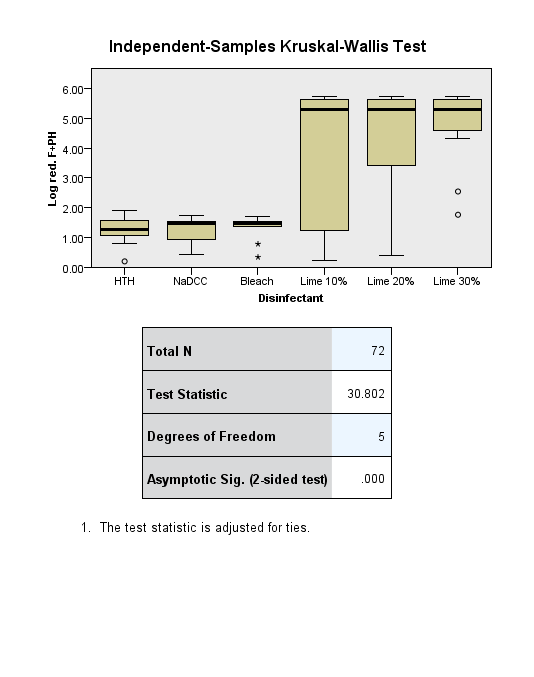
**

**
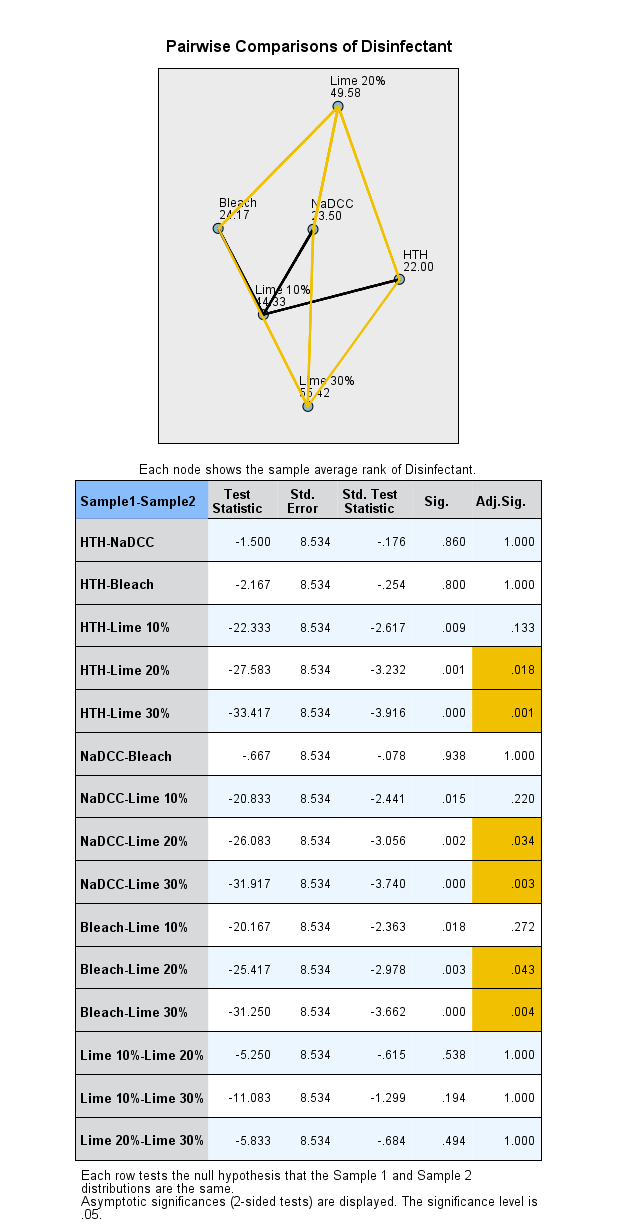
**

**
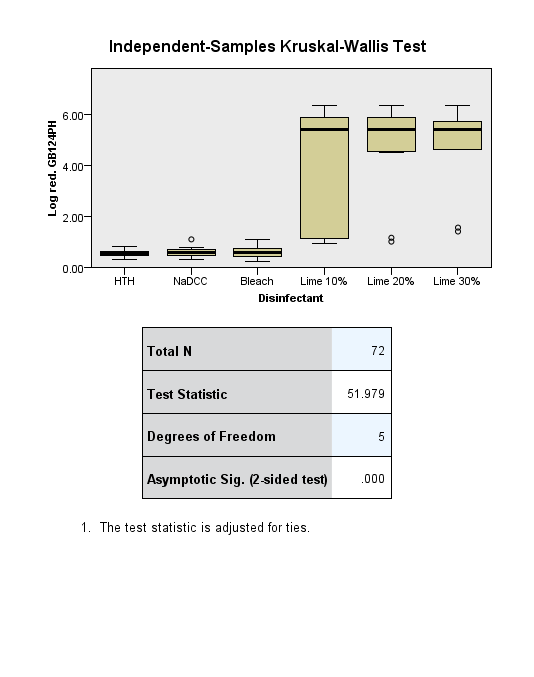
**

**
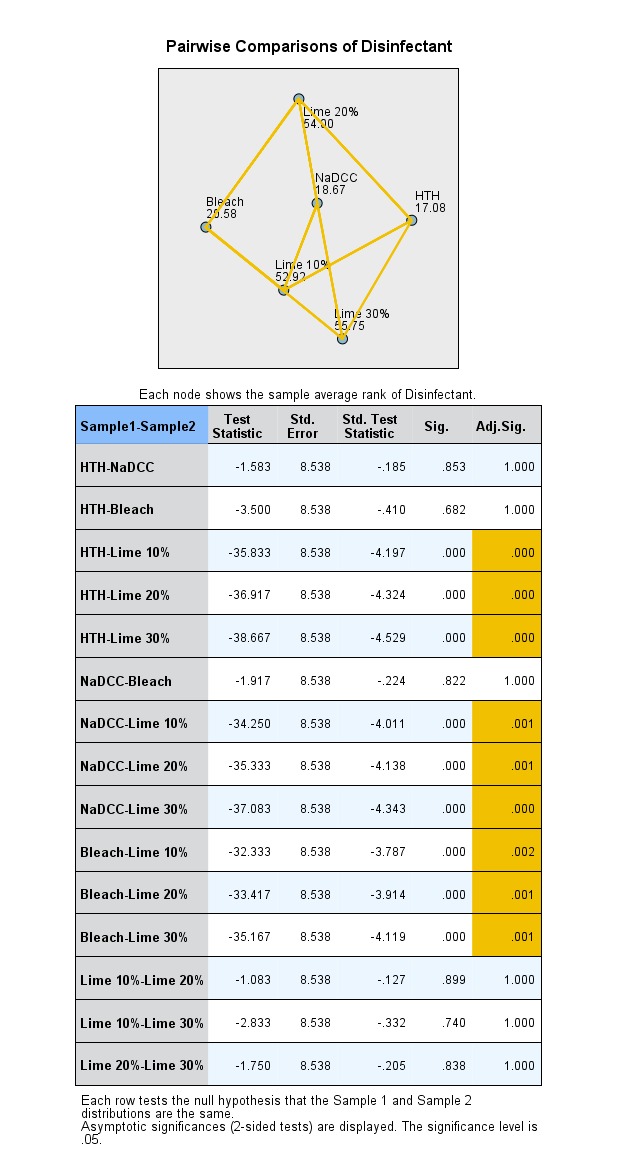
**
