# Supplementary material for: Assessment of recommended approaches for containment and safe handling of human excreta in emergency settings
Source: PLoS One. 2018 Jul 26;13(7):e0201344. doi: 10.1371/journal.pone.0201344 (PMC6062132; doi:10.1371/journal.pone.0201344)

# S4 File Man-Witney statistical tests comparing each approach efficacy according to contact time (Ct) and mixing treatment

Hypothesises:

- The distribution of log10 reduction of microorganism is the same across categories of ‘contact time’
- The distribution of log10 reduction of microorganism is the same across categories of ‘mixed/non-mixed disinfectant/excreta matrix

## S5 a. Disinfectant: HTH

### Contac time

### Mixed vs. Non-mixed

## S5 b. Disinfectant: NADCC

### Contact time

### Mixed vs. Non-mixed

## S5 c. Disinfectant: Bleach

### Contact time

### Mixed vs Non-mixed

## S5 d. Disinfectant: Lime 10%

### Contact time

### Mixed vs Non-mixed

**Intestina enterococci**


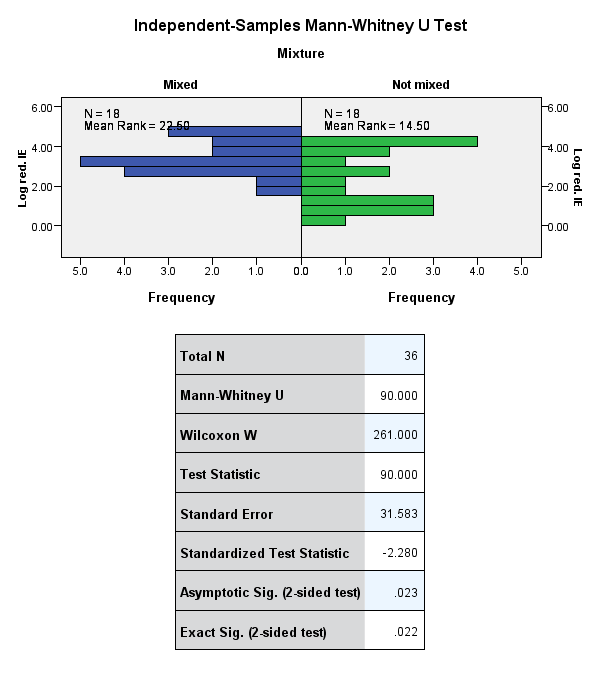


**F+ Specific phages**


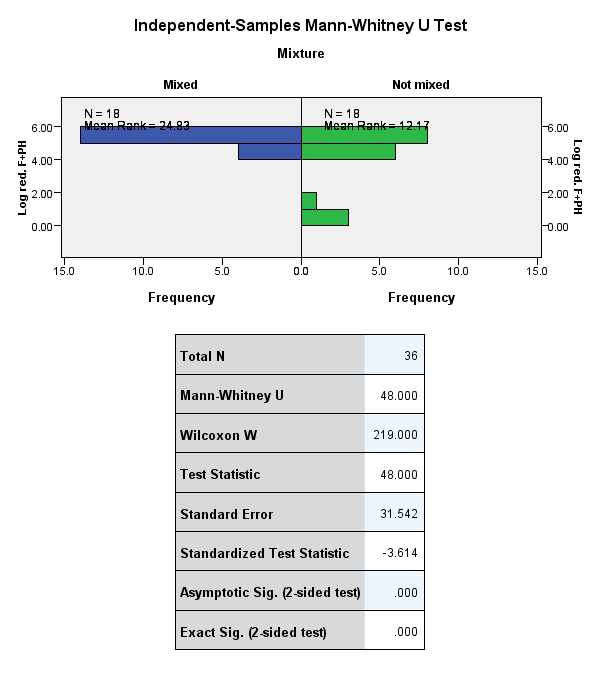


## S5 e. Disinfectant: Lime 20%

### Contact time

### Mixed vs Non-mixed

**Faecal coliforms**


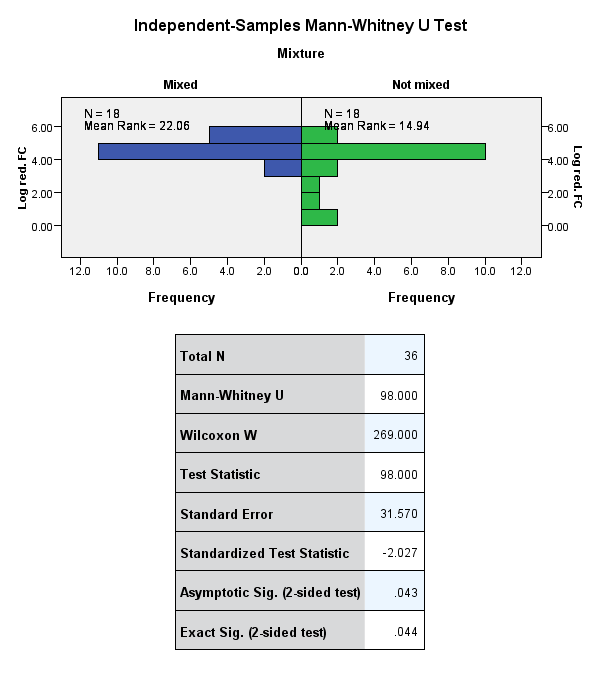


**F+ specific phages**


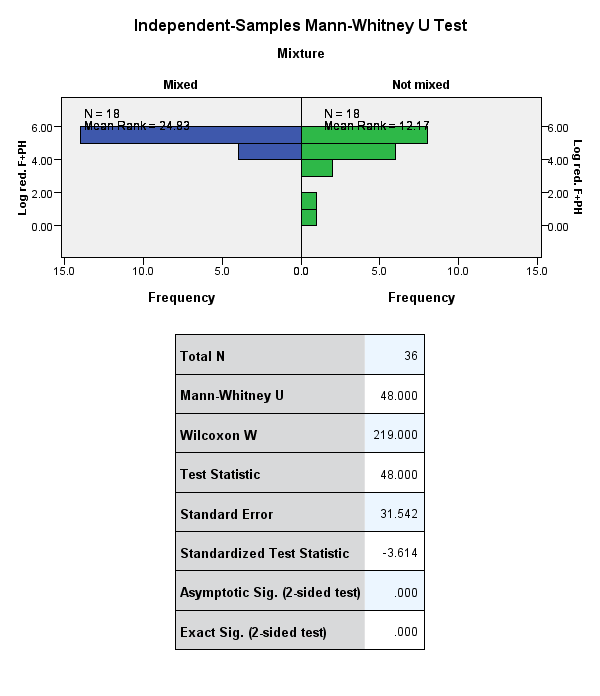


## S5 f. Disinfectant: Lime 30%

### Contact time

### Mixed vs Non-mixed

**Faecal coliforms**


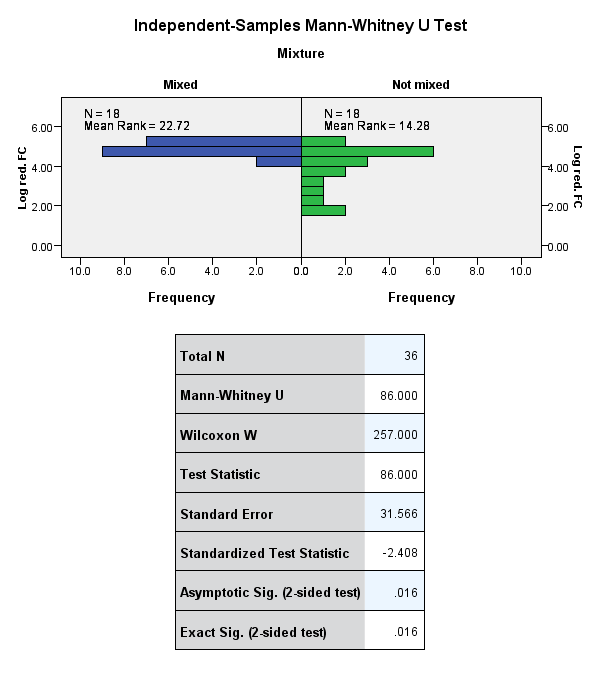


**Intestinal enterococci**


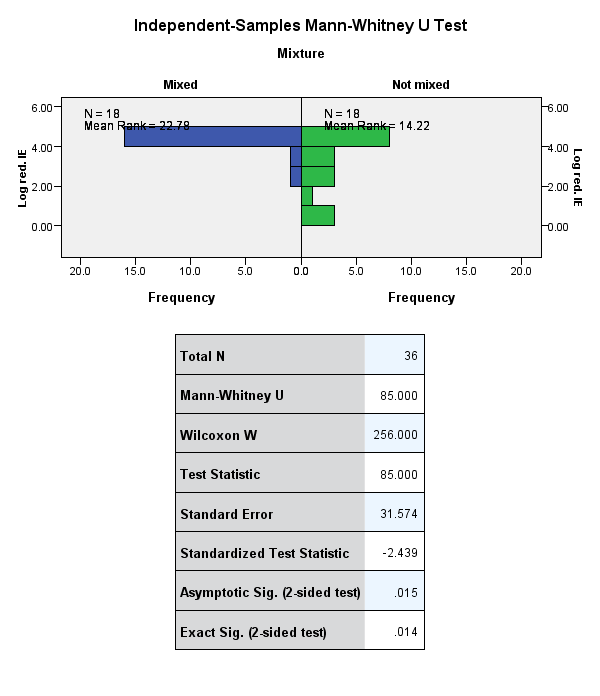


**F+ specific phages**


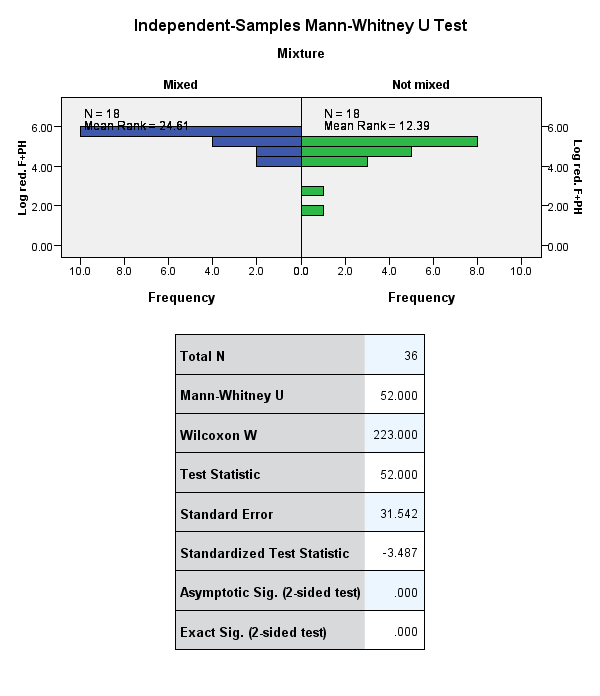

Supplement: S4 File — (DOCX) [file pone.0201344.s004.docx]
