# Supplementary material for: Assessment of recommended approaches for containment and safe handling of human excreta in emergency settings
Source: PLoS One. 2018 Jul 26;13(7):e0201344. doi: 10.1371/journal.pone.0201344 (PMC6062132; doi:10.1371/journal.pone.0201344)
Supplement: S5 File — (DOCX) [file pone.0201344.s005.docx]

**S5 File. Statistics tests related to chlorine demand**

| **ANOVA** | | | | | | |
| --- | --- | --- | --- | --- | --- | --- |
|  | | Sum of Squares | df | Mean Square | F | Sig. |
| Chlorinem0 | Between Groups | 4062.500 | 2 | 2031.250 | .299 | .744 |
|  | Within Groups | 224368.250 | 33 | 6799.038 |  |  |
|  | Total | 228430.750 | 35 |  |  |  |
| Chlorine10m10 | Between Groups | .154 | 2 | .077 | .125 | .883 |
|  | Within Groups | 20.276 | 33 | .614 |  |  |
|  | Total | 20.430 | 35 |  |  |  |
| Chlorine10m20 | Between Groups | .101 | 2 | .050 | .179 | .837 |
|  | Within Groups | 9.248 | 33 | .280 |  |  |
|  | Total | 9.349 | 35 |  |  |  |

| **Multiple Comparisons** | | | | | | | |
| --- | --- | --- | --- | --- | --- | --- | --- |
| Tukey HSD | | | | | | | |
| Dependent Variable | (I) Disinfectant | (J) Disinfectant | Mean Difference (I-J) | Std. Error | Sig. | 95% Confidence Interval | |
|  |  |  |  |  |  | Lower Bound | Upper Bound |
| Chlorinem0 | HTH | NaDCC | -25.00000 | 33.66263 | .740 | -107.6012 | 57.6012 |
|  |  | Bleach | -6.25000 | 33.66263 | .981 | -88.8512 | 76.3512 |
|  | NaDCC | HTH | 25.00000 | 33.66263 | .740 | -57.6012 | 107.6012 |
|  |  | Bleach | 18.75000 | 33.66263 | .844 | -63.8512 | 101.3512 |
|  | Bleach | HTH | 6.25000 | 33.66263 | .981 | -76.3512 | 88.8512 |
|  |  | NaDCC | -18.75000 | 33.66263 | .844 | -101.3512 | 63.8512 |
| Chlorine10m10 | HTH | NaDCC | -.10000 | .32000 | .948 | -.8852 | .6852 |
|  |  | Bleach | -.15833 | .32000 | .874 | -.9436 | .6269 |
|  | NaDCC | HTH | .10000 | .32000 | .948 | -.6852 | .8852 |
|  |  | Bleach | -.05833 | .32000 | .982 | -.8436 | .7269 |
|  | Bleach | HTH | .15833 | .32000 | .874 | -.6269 | .9436 |
|  |  | NaDCC | .05833 | .32000 | .982 | -.7269 | .8436 |
| Chlorine10m20 | HTH | NaDCC | .03333 | .21612 | .987 | -.4970 | .5637 |
|  |  | Bleach | .12500 | .21612 | .833 | -.4053 | .6553 |
|  | NaDCC | HTH | -.03333 | .21612 | .987 | -.5637 | .4970 |
|  |  | Bleach | .09167 | .21612 | .906 | -.4387 | .6220 |
|  | Bleach | HTH | -.12500 | .21612 | .833 | -.6553 | .4053 |
|  |  | NaDCC | -.09167 | .21612 | .906 | -.6220 | .4387 |

| **Chlorinem0** | | |
| --- | --- | --- |
| Tukey HSD^a^ | | |
| Disinfectant | N | Subset for alpha = 0.05 |
|  |  | 1 |
| HTH | 12 | 200.5000 |
| Bleach | 12 | 206.7500 |
| NaDCC | 12 | 225.5000 |
| Sig. |  | .740 |
| Means for groups in homogeneous subsets are displayed. | | |
| a. Uses Harmonic Mean Sample Size = 12.000. | | |

| **Chlorine10m10** | | |
| --- | --- | --- |
| Tukey HSD^a^ | | |
| Disinfectant | N | Subset for alpha = 0.05 |
|  |  | 1 |
| HTH | 12 | 1.7167 |
| NaDCC | 12 | 1.8167 |
| Bleach | 12 | 1.8750 |
| Sig. |  | .874 |
| Means for groups in homogeneous subsets are displayed. | | |
| a. Uses Harmonic Mean Sample Size = 12.000. | | |

| **Chlorine10m20** | | |
| --- | --- | --- |
| Tukey HSD^a^ | | |
| Disinfectant | N | Subset for alpha = 0.05 |
|  |  | 1 |
| Bleach | 12 | .7833 |
| NaDCC | 12 | .8750 |
| HTH | 12 | .9083 |
| Sig. |  | .833 |
| Means for groups in homogeneous subsets are displayed. | | |
| a. Uses Harmonic Mean Sample Size = 12.000. | | |

HTH

| **Group Statistics** | | | | | |
| --- | --- | --- | --- | --- | --- |
|  | Contact Time | N | Mean | Std. Deviation | Std. Error Mean |
| Chlorinem0 | 15 minutes | 6 | 213.5000 | 90.97197 | 37.13915 |
|  | 30 minutes | 6 | 187.5000 | 76.15182 | 31.08885 |
| Chlorine10m10 | 15 minutes | 6 | 2.3167 | .49160 | .20069 |
|  | 30 minutes | 6 | 1.1167 | .77309 | .31561 |
| Chlorine10m20 | 15 minutes | 6 | .9833 | .75476 | .30813 |
|  | 30 minutes | 6 | .8333 | .50859 | .20763 |

| **Independent Samples Test** | | | | | | | | | | |
| --- | --- | --- | --- | --- | --- | --- | --- | --- | --- | --- |
|  | | Levene's Test for Equality of Variances | | t-test for Equality of Means | | | | | | |
|  |  | F | Sig. | t | df | Sig. (2-tailed) | Mean Difference | Std. Error Difference | 95% Confidence Interval of the Difference | |
|  |  |  |  |  |  |  |  |  | Lower | Upper |
| Chlorinem0 | Equal variances assumed | .244 | .632 | .537 | 10 | .603 | 26.00000 | 48.43380 | -81.91724 | 133.91724 |
|  | Equal variances not assumed |  |  | .537 | 9.700 | .603 | 26.00000 | 48.43380 | -82.37193 | 134.37193 |
| Chlorine10m10 | Equal variances assumed | 1.581 | .237 | 3.208 | 10 | .009 | 1.20000 | .37402 | .36664 | 2.03336 |
|  | Equal variances not assumed |  |  | 3.208 | 8.475 | .012 | 1.20000 | .37402 | .34586 | 2.05414 |
| Chlorine10m20 | Equal variances assumed | 1.233 | .293 | .404 | 10 | .695 | .15000 | .37156 | -.67788 | .97788 |
|  | Equal variances not assumed |  |  | .404 | 8.765 | .696 | .15000 | .37156 | -.69398 | .99398 |

NADCC

| **Group Statistics** | | | | | |
| --- | --- | --- | --- | --- | --- |
|  | Contact Time | N | Mean | Std. Deviation | Std. Error Mean |
| Chlorinem0 | 15 minutes | 6 | 241.3333 | 85.94572 | 35.08719 |
|  | 30 minutes | 6 | 209.6667 | 77.12241 | 31.48509 |
| Chlorine10m10 | 15 minutes | 6 | 2.0167 | 1.00083 | .40859 |
|  | 30 minutes | 6 | 1.6167 | .42622 | .17401 |
| Chlorine10m20 | 15 minutes | 6 | .8667 | .43205 | .17638 |
|  | 30 minutes | 6 | .8833 | .47504 | .19394 |

| **Independent Samples Test** | | | | | | | | | | |
| --- | --- | --- | --- | --- | --- | --- | --- | --- | --- | --- |
|  | | Levene's Test for Equality of Variances | | t-test for Equality of Means | | | | | | |
|  |  | F | Sig. | t | df | Sig. (2-tailed) | Mean Difference | Std. Error Difference | 95% Confidence Interval of the Difference | |
|  |  |  |  |  |  |  |  |  | Lower | Upper |
| Chlorinem0 | Equal variances assumed | .342 | .571 | .672 | 10 | .517 | 31.66667 | 47.14257 | -73.37353 | 136.70687 |
|  | Equal variances not assumed |  |  | .672 | 9.885 | .517 | 31.66667 | 47.14257 | -73.53953 | 136.87287 |
| Chlorine10m10 | Equal variances assumed | 5.537 | .040 | .901 | 10 | .389 | .40000 | .44410 | -.58951 | 1.38951 |
|  | Equal variances not assumed |  |  | .901 | 6.756 | .399 | .40000 | .44410 | -.65786 | 1.45786 |
| Chlorine10m20 | Equal variances assumed | .166 | .692 | -.064 | 10 | .951 | -.01667 | .26215 | -.60077 | .56744 |
|  | Equal variances not assumed |  |  | -.064 | 9.911 | .951 | -.01667 | .26215 | -.60148 | .56815 |

Bleach

| **Group Statistics** | | | | | |
| --- | --- | --- | --- | --- | --- |
|  | Contact Time | N | Mean | Std. Deviation | Std. Error Mean |
| Chlorinem0 | 15 minutes | 6 | 208.6667 | 86.94749 | 35.49617 |
|  | 30 minutes | 6 | 204.8333 | 94.27707 | 38.48845 |
| Chlorine10m10 | 15 minutes | 6 | 2.0667 | .81404 | .33233 |
|  | 30 minutes | 6 | 1.6833 | .56716 | .23154 |
| Chlorine10m20 | 15 minutes | 6 | .7667 | .53914 | .22010 |
|  | 30 minutes | 6 | .8000 | .55136 | .22509 |

| **Independent Samples Test** | | | | | | | | | | |
| --- | --- | --- | --- | --- | --- | --- | --- | --- | --- | --- |
|  | | Levene's Test for Equality of Variances | | t-test for Equality of Means | | | | | | |
|  |  | F | Sig. | t | df | Sig. (2-tailed) | Mean Difference | Std. Error Difference | 95% Confidence Interval of the Difference | |
|  |  |  |  |  |  |  |  |  | Lower | Upper |
| Chlorinem0 | Equal variances assumed | .000 | .993 | .073 | 10 | .943 | 3.83333 | 52.35780 | -112.82711 | 120.49377 |
|  | Equal variances not assumed |  |  | .073 | 9.935 | .943 | 3.83333 | 52.35780 | -112.93032 | 120.59699 |
| Chlorine10m10 | Equal variances assumed | .076 | .789 | .946 | 10 | .366 | .38333 | .40504 | -.51915 | 1.28581 |
|  | Equal variances not assumed |  |  | .946 | 8.928 | .369 | .38333 | .40504 | -.53405 | 1.30071 |
| Chlorine10m20 | Equal variances assumed | .004 | .953 | -.106 | 10 | .918 | -.03333 | .31482 | -.73479 | .66813 |
|  | Equal variances not assumed |  |  | -.106 | 9.995 | .918 | -.03333 | .31482 | -.73484 | .66818 |
