# Supplementary material for: Assessment of recommended approaches for containment and safe handling of human excreta in emergency settings
Source: PLoS One. 2018 Jul 26;13(7):e0201344. doi: 10.1371/journal.pone.0201344 (PMC6062132; doi:10.1371/journal.pone.0201344)
Supplement: S6 File — (DOCX) [file pone.0201344.s006.docx]

**S10. Statistics tests related to pH**

**T-Test pH levels Mixed vs non-mixed**

[DataSet1] C:\Users\dds12\Dropbox\BUCKET PAPER\Bucket paper SPSS + Excell\PH for support information\HTH pH Matrix 0%.sav

| **Group Statistics** | | | | | |
| --- | --- | --- | --- | --- | --- |
|  | Mixture | N | Mean | Std. Deviation | Std. Error Mean |
| pH | Mixed | 6 | 6.1917 | .23532 | .09607 |
|  | Not mixed | 6 | 5.8783 | .06178 | .02522 |

| **Independent Samples Test** | | | | | | | | | | |
| --- | --- | --- | --- | --- | --- | --- | --- | --- | --- | --- |
|  | | Levene's Test for Equality of Variances | | t-test for Equality of Means | | | | | | |
|  |  | F | Sig. | t | df | Sig. (2-tailed) | Mean Difference | Std. Error Difference | 95% Confidence Interval of the Difference | |
|  |  |  |  |  |  |  |  |  | Lower | Upper |
| pH | Equal variances assumed | 6.161 | .032 | 3.155 | 10 | .010 | .31333 | .09933 | .09202 | .53464 |
|  | Equal variances not assumed |  |  | 3.155 | 5.686 | .021 | .31333 | .09933 | .06700 | .55967 |

**T-Test**

| **Notes** | | |
| --- | --- | --- |
| Output Created | | 23-JUN-2017 15:18:17 |
| Comments | |  |
| Input | Data | C:\Users\dds12\Dropbox\BUCKET PAPER\Bucket paper SPSS + Excell\PH for support information\HTH pH Matrix 10%.sav |
|  | Active Dataset | DataSet2 |
|  | Filter | <none> |
|  | Weight | <none> |
|  | Split File | <none> |
|  | N of Rows in Working Data File | 13 |
| Missing Value Handling | Definition of Missing | User defined missing values are treated as missing. |
|  | Cases Used | Statistics for each analysis are based on the cases with no missing or out-of-range data for any variable in the analysis. |
| Syntax | | T-TEST GROUPS=Mixture(1 2)  /MISSING=ANALYSIS  /VARIABLES=pH  /CRITERIA=CI(.95). |
| Resources | Processor Time | 00:00:00.00 |
|  | Elapsed Time | 00:00:00.00 |

[DataSet2] C:\Users\dds12\Dropbox\BUCKET PAPER\Bucket paper SPSS + Excell\PH for support information\HTH pH Matrix 10%.sav

| **Group Statistics** | | | | | |
| --- | --- | --- | --- | --- | --- |
|  | Mixture | N | Mean | Std. Deviation | Std. Error Mean |
| pH | Mixed | 6 | 6.1550 | .48472 | .19788 |
|  | Not mixed | 6 | 6.4700 | .48033 | .19610 |

| **Independent Samples Test** | | | | | | | | | | |
| --- | --- | --- | --- | --- | --- | --- | --- | --- | --- | --- |
|  | | Levene's Test for Equality of Variances | | t-test for Equality of Means | | | | | | |
|  |  | F | Sig. | t | df | Sig. (2-tailed) | Mean Difference | Std. Error Difference | 95% Confidence Interval of the Difference | |
|  |  |  |  |  |  |  |  |  | Lower | Upper |
| pH | Equal variances assumed | .001 | .972 | -1.131 | 10 | .285 | -.31500 | .27859 | -.93573 | .30573 |
|  | Equal variances not assumed |  |  | -1.131 | 9.999 | .285 | -.31500 | .27859 | -.93574 | .30574 |

**T-Test**

| **Notes** | | |
| --- | --- | --- |
| Output Created | | 23-JUN-2017 15:19:10 |
| Comments | |  |
| Input | Data | C:\Users\dds12\Dropbox\BUCKET PAPER\Bucket paper SPSS + Excell\PH for support information\HTH pH Matrix 20%.sav |
|  | Active Dataset | DataSet3 |
|  | Filter | <none> |
|  | Weight | <none> |
|  | Split File | <none> |
|  | N of Rows in Working Data File | 13 |
| Missing Value Handling | Definition of Missing | User defined missing values are treated as missing. |
|  | Cases Used | Statistics for each analysis are based on the cases with no missing or out-of-range data for any variable in the analysis. |
| Syntax | | T-TEST GROUPS=Mixture(1 2)  /MISSING=ANALYSIS  /VARIABLES=pH  /CRITERIA=CI(.95). |
| Resources | Processor Time | 00:00:00.02 |
|  | Elapsed Time | 00:00:00.01 |

[DataSet3] C:\Users\dds12\Dropbox\BUCKET PAPER\Bucket paper SPSS + Excell\PH for support information\HTH pH Matrix 20%.sav

| **Group Statistics** | | | | | |
| --- | --- | --- | --- | --- | --- |
|  | Mixture | N | Mean | Std. Deviation | Std. Error Mean |
| pH | Mixed | 6 | 6.3683 | .64750 | .26434 |
|  | Not mixed | 6 | 6.1983 | .69600 | .28414 |

| **Independent Samples Test** | | | | | | | | | | |
| --- | --- | --- | --- | --- | --- | --- | --- | --- | --- | --- |
|  | | Levene's Test for Equality of Variances | | t-test for Equality of Means | | | | | | |
|  |  | F | Sig. | t | df | Sig. (2-tailed) | Mean Difference | Std. Error Difference | 95% Confidence Interval of the Difference | |
|  |  |  |  |  |  |  |  |  | Lower | Upper |
| pH | Equal variances assumed | .289 | .603 | .438 | 10 | .671 | .17000 | .38809 | -.69471 | 1.03471 |
|  | Equal variances not assumed |  |  | .438 | 9.948 | .671 | .17000 | .38809 | -.69532 | 1.03532 |

**T-Test**

| **Notes** | | |
| --- | --- | --- |
| Output Created | | 23-JUN-2017 15:19:35 |
| Comments | |  |
| Input | Data | C:\Users\dds12\Dropbox\BUCKET PAPER\Bucket paper SPSS + Excell\PH for support information\NADCCpH matrix 0%.sav |
|  | Active Dataset | DataSet4 |
|  | Filter | <none> |
|  | Weight | <none> |
|  | Split File | <none> |
|  | N of Rows in Working Data File | 13 |
| Missing Value Handling | Definition of Missing | User defined missing values are treated as missing. |
|  | Cases Used | Statistics for each analysis are based on the cases with no missing or out-of-range data for any variable in the analysis. |
| Syntax | | T-TEST GROUPS=Mixture(1 2)  /MISSING=ANALYSIS  /VARIABLES=pH  /CRITERIA=CI(.95). |
| Resources | Processor Time | 00:00:00.00 |
|  | Elapsed Time | 00:00:00.01 |

[DataSet4] C:\Users\dds12\Dropbox\BUCKET PAPER\Bucket paper SPSS + Excell\PH for support information\NADCCpH matrix 0%.sav

| **Group Statistics** | | | | | |
| --- | --- | --- | --- | --- | --- |
|  | Mixture | N | Mean | Std. Deviation | Std. Error Mean |
| pH | Mixed | 6 | 5.9117 | .11179 | .04564 |
|  | Not mixed | 6 | 5.9483 | .16253 | .06635 |

| **Independent Samples Test** | | | | | | | | | | |
| --- | --- | --- | --- | --- | --- | --- | --- | --- | --- | --- |
|  | | Levene's Test for Equality of Variances | | t-test for Equality of Means | | | | | | |
|  |  | F | Sig. | t | df | Sig. (2-tailed) | Mean Difference | Std. Error Difference | 95% Confidence Interval of the Difference | |
|  |  |  |  |  |  |  |  |  | Lower | Upper |
| pH | Equal variances assumed | 1.333 | .275 | -.455 | 10 | .659 | -.03667 | .08053 | -.21611 | .14277 |
|  | Equal variances not assumed |  |  | -.455 | 8.866 | .660 | -.03667 | .08053 | -.21927 | .14593 |

**T-Test**

| **Notes** | | |
| --- | --- | --- |
| Output Created | | 23-JUN-2017 15:20:11 |
| Comments | |  |
| Input | Data | C:\Users\dds12\Dropbox\BUCKET PAPER\Bucket paper SPSS + Excell\PH for support information\NADCCpH. Matrix 10sav.sav |
|  | Active Dataset | DataSet5 |
|  | Filter | <none> |
|  | Weight | <none> |
|  | Split File | <none> |
|  | N of Rows in Working Data File | 13 |
| Missing Value Handling | Definition of Missing | User defined missing values are treated as missing. |
|  | Cases Used | Statistics for each analysis are based on the cases with no missing or out-of-range data for any variable in the analysis. |
| Syntax | | T-TEST GROUPS=Mixture(1 2)  /MISSING=ANALYSIS  /VARIABLES=pH  /CRITERIA=CI(.95). |
| Resources | Processor Time | 00:00:00.00 |
|  | Elapsed Time | 00:00:00.00 |

[DataSet5] C:\Users\dds12\Dropbox\BUCKET PAPER\Bucket paper SPSS + Excell\PH for support information\NADCCpH. Matrix 10sav.sav

| **Group Statistics** | | | | | |
| --- | --- | --- | --- | --- | --- |
|  | Mixture | N | Mean | Std. Deviation | Std. Error Mean |
| pH | Mixed | 6 | 5.8417 | .17736 | .07241 |
|  | Not mixed | 6 | 5.9367 | .18981 | .07749 |

| **Independent Samples Test** | | | | | | | | | | |
| --- | --- | --- | --- | --- | --- | --- | --- | --- | --- | --- |
|  | | Levene's Test for Equality of Variances | | t-test for Equality of Means | | | | | | |
|  |  | F | Sig. | t | df | Sig. (2-tailed) | Mean Difference | Std. Error Difference | 95% Confidence Interval of the Difference | |
|  |  |  |  |  |  |  |  |  | Lower | Upper |
| pH | Equal variances assumed | .109 | .748 | -.896 | 10 | .391 | -.09500 | .10605 | -.33130 | .14130 |
|  | Equal variances not assumed |  |  | -.896 | 9.954 | .392 | -.09500 | .10605 | -.33145 | .14145 |

**T-Test**

| **Notes** | | |
| --- | --- | --- |
| Output Created | | 23-JUN-2017 15:21:06 |
| Comments | |  |
| Input | Data | C:\Users\dds12\Dropbox\BUCKET PAPER\Bucket paper SPSS + Excell\PH for support information\NADCCpH. Matrix 20.sav |
|  | Active Dataset | DataSet6 |
|  | Filter | <none> |
|  | Weight | <none> |
|  | Split File | <none> |
|  | N of Rows in Working Data File | 13 |
| Missing Value Handling | Definition of Missing | User defined missing values are treated as missing. |
|  | Cases Used | Statistics for each analysis are based on the cases with no missing or out-of-range data for any variable in the analysis. |
| Syntax | | T-TEST GROUPS=Mixture(1 2)  /MISSING=ANALYSIS  /VARIABLES=pH  /CRITERIA=CI(.95). |
| Resources | Processor Time | 00:00:00.00 |
|  | Elapsed Time | 00:00:00.01 |

[DataSet6] C:\Users\dds12\Dropbox\BUCKET PAPER\Bucket paper SPSS + Excell\PH for support information\NADCCpH. Matrix 20.sav

| **Group Statistics** | | | | | |
| --- | --- | --- | --- | --- | --- |
|  | Mixture | N | Mean | Std. Deviation | Std. Error Mean |
| pH | Mixed | 6 | 6.3000 | .51248 | .20922 |
|  | Not mixed | 6 | 6.2183 | .59881 | .24446 |

| **Independent Samples Test** | | | | | | | | | | |
| --- | --- | --- | --- | --- | --- | --- | --- | --- | --- | --- |
|  | | Levene's Test for Equality of Variances | | t-test for Equality of Means | | | | | | |
|  |  | F | Sig. | t | df | Sig. (2-tailed) | Mean Difference | Std. Error Difference | 95% Confidence Interval of the Difference | |
|  |  |  |  |  |  |  |  |  | Lower | Upper |
| pH | Equal variances assumed | .131 | .725 | .254 | 10 | .805 | .08167 | .32177 | -.63528 | .79862 |
|  | Equal variances not assumed |  |  | .254 | 9.767 | .805 | .08167 | .32177 | -.63761 | .80094 |

**T-Test**

| **Notes** | | |
| --- | --- | --- |
| Output Created | | 23-JUN-2017 15:22:46 |
| Comments | |  |
| Input | Data | C:\Users\dds12\Dropbox\BUCKET PAPER\Bucket paper SPSS + Excell\PH for support information\Bleach pH.Matrix 0%.sav |
|  | Active Dataset | DataSet7 |
|  | Filter | <none> |
|  | Weight | <none> |
|  | Split File | <none> |
|  | N of Rows in Working Data File | 12 |
| Missing Value Handling | Definition of Missing | User defined missing values are treated as missing. |
|  | Cases Used | Statistics for each analysis are based on the cases with no missing or out-of-range data for any variable in the analysis. |
| Syntax | | T-TEST GROUPS=Mixture(1 2)  /MISSING=ANALYSIS  /VARIABLES=pH  /CRITERIA=CI(.95). |
| Resources | Processor Time | 00:00:00.00 |
|  | Elapsed Time | 00:00:00.00 |

[DataSet7] C:\Users\dds12\Dropbox\BUCKET PAPER\Bucket paper SPSS + Excell\PH for support information\Bleach pH.Matrix 0%.sav

| **Group Statistics** | | | | | |
| --- | --- | --- | --- | --- | --- |
|  | Mixture | N | Mean | Std. Deviation | Std. Error Mean |
| pH | Mixed | 6 | 6.6967 | 1.02549 | .41865 |
|  | Not mixed | 6 | 7.8000 | .26077 | .10646 |

| **Independent Samples Test** | | | | | | | | | | |
| --- | --- | --- | --- | --- | --- | --- | --- | --- | --- | --- |
|  | | Levene's Test for Equality of Variances | | t-test for Equality of Means | | | | | | |
|  |  | F | Sig. | t | df | Sig. (2-tailed) | Mean Difference | Std. Error Difference | 95% Confidence Interval of the Difference | |
|  |  |  |  |  |  |  |  |  | Lower | Upper |
| pH | Equal variances assumed | 20.998 | .001 | -2.554 | 10 | .029 | -1.10333 | .43198 | -2.06584 | -.14083 |
|  | Equal variances not assumed |  |  | -2.554 | 5.644 | .046 | -1.10333 | .43198 | -2.17672 | -.02995 |

**T-Test**

| **Notes** | | |
| --- | --- | --- |
| Output Created | | 23-JUN-2017 15:23:44 |
| Comments | |  |
| Input | Data | C:\Users\dds12\Dropbox\BUCKET PAPER\Bucket paper SPSS + Excell\PH for support information\Bleach pH.Matrix 10%.sav |
|  | Active Dataset | DataSet8 |
|  | Filter | <none> |
|  | Weight | <none> |
|  | Split File | <none> |
|  | N of Rows in Working Data File | 12 |
| Missing Value Handling | Definition of Missing | User defined missing values are treated as missing. |
|  | Cases Used | Statistics for each analysis are based on the cases with no missing or out-of-range data for any variable in the analysis. |
| Syntax | | T-TEST GROUPS=Mixture(1 2)  /MISSING=ANALYSIS  /VARIABLES=pH  /CRITERIA=CI(.95). |
| Resources | Processor Time | 00:00:00.00 |
|  | Elapsed Time | 00:00:00.00 |

[DataSet8] C:\Users\dds12\Dropbox\BUCKET PAPER\Bucket paper SPSS + Excell\PH for support information\Bleach pH.Matrix 10%.sav

| **Group Statistics** | | | | | |
| --- | --- | --- | --- | --- | --- |
|  | Mixture | N | Mean | Std. Deviation | Std. Error Mean |
| pH | Mixed | 6 | 6.3117 | .09390 | .03833 |
|  | Not mixed | 6 | 6.7000 | .38987 | .15916 |

| **Independent Samples Test** | | | | | | | | | | |
| --- | --- | --- | --- | --- | --- | --- | --- | --- | --- | --- |
|  | | Levene's Test for Equality of Variances | | t-test for Equality of Means | | | | | | |
|  |  | F | Sig. | t | df | Sig. (2-tailed) | Mean Difference | Std. Error Difference | 95% Confidence Interval of the Difference | |
|  |  |  |  |  |  |  |  |  | Lower | Upper |
| pH | Equal variances assumed | 19.391 | .001 | -2.372 | 10 | .039 | -.38833 | .16372 | -.75311 | -.02355 |
|  | Equal variances not assumed |  |  | -2.372 | 5.578 | .059 | -.38833 | .16372 | -.79639 | .01972 |

**T-Test**

| **Notes** | | |
| --- | --- | --- |
| Output Created | | 23-JUN-2017 15:24:17 |
| Comments | |  |
| Input | Data | C:\Users\dds12\Dropbox\BUCKET PAPER\Bucket paper SPSS + Excell\PH for support information\Bleach pH.Matrix 20%.sav |
|  | Active Dataset | DataSet9 |
|  | Filter | <none> |
|  | Weight | <none> |
|  | Split File | <none> |
|  | N of Rows in Working Data File | 12 |
| Missing Value Handling | Definition of Missing | User defined missing values are treated as missing. |
|  | Cases Used | Statistics for each analysis are based on the cases with no missing or out-of-range data for any variable in the analysis. |
| Syntax | | T-TEST GROUPS=Mixture(1 2)  /MISSING=ANALYSIS  /VARIABLES=pH  /CRITERIA=CI(.95). |
| Resources | Processor Time | 00:00:00.00 |
|  | Elapsed Time | 00:00:00.00 |

[DataSet9] C:\Users\dds12\Dropbox\BUCKET PAPER\Bucket paper SPSS + Excell\PH for support information\Bleach pH.Matrix 20%.sav

| **Group Statistics** | | | | | |
| --- | --- | --- | --- | --- | --- |
|  | Mixture | N | Mean | Std. Deviation | Std. Error Mean |
| pH | Mixed | 6 | 6.8217 | .63351 | .25863 |
|  | Not mixed | 6 | 6.8167 | .47924 | .19565 |

| **Independent Samples Test** | | | | | | | | | | |
| --- | --- | --- | --- | --- | --- | --- | --- | --- | --- | --- |
|  | | Levene's Test for Equality of Variances | | t-test for Equality of Means | | | | | | |
|  |  | F | Sig. | t | df | Sig. (2-tailed) | Mean Difference | Std. Error Difference | 95% Confidence Interval of the Difference | |
|  |  |  |  |  |  |  |  |  | Lower | Upper |
| pH | Equal variances assumed | .594 | .459 | .015 | 10 | .988 | .00500 | .32429 | -.71757 | .72757 |
|  | Equal variances not assumed |  |  | .015 | 9.311 | .988 | .00500 | .32429 | -.72489 | .73489 |

**T-Test**

| **Notes** | | |
| --- | --- | --- |
| Output Created | | 23-JUN-2017 15:25:54 |
| Comments | |  |
| Input | Data | C:\Users\dds12\Dropbox\BUCKET PAPER\Bucket paper SPSS + Excell\PH for support information\Lime 10pH. Matrix 0% sav.sav |
|  | Active Dataset | DataSet10 |
|  | Filter | <none> |
|  | Weight | <none> |
|  | Split File | <none> |
|  | N of Rows in Working Data File | 12 |
| Missing Value Handling | Definition of Missing | User defined missing values are treated as missing. |
|  | Cases Used | Statistics for each analysis are based on the cases with no missing or out-of-range data for any variable in the analysis. |
| Syntax | | T-TEST GROUPS=Mixture(1 2)  /MISSING=ANALYSIS  /VARIABLES=pH  /CRITERIA=CI(.95). |
| Resources | Processor Time | 00:00:00.02 |
|  | Elapsed Time | 00:00:00.01 |

[DataSet10] C:\Users\dds12\Dropbox\BUCKET PAPER\Bucket paper SPSS + Excell\PH for support information\Lime 10pH. Matrix 0% sav.sav

| **Group Statistics** | | | | | |
| --- | --- | --- | --- | --- | --- |
|  | Mixture | N | Mean | Std. Deviation | Std. Error Mean |
| pH | Mixed | 6 | 12.9200 | .32912 | .13436 |
|  | Not mixed | 6 | 12.7350 | .16233 | .06627 |

| **Independent Samples Test** | | | | | | | | | | |
| --- | --- | --- | --- | --- | --- | --- | --- | --- | --- | --- |
|  | | Levene's Test for Equality of Variances | | t-test for Equality of Means | | | | | | |
|  |  | F | Sig. | t | df | Sig. (2-tailed) | Mean Difference | Std. Error Difference | 95% Confidence Interval of the Difference | |
|  |  |  |  |  |  |  |  |  | Lower | Upper |
| pH | Equal variances assumed | 3.175 | .105 | 1.235 | 10 | .245 | .18500 | .14982 | -.14881 | .51881 |
|  | Equal variances not assumed |  |  | 1.235 | 7.297 | .255 | .18500 | .14982 | -.16636 | .53636 |

**T-Test**

| **Notes** | | |
| --- | --- | --- |
| Output Created | | 23-JUN-2017 15:26:17 |
| Comments | |  |
| Input | Data | C:\Users\dds12\Dropbox\BUCKET PAPER\Bucket paper SPSS + Excell\PH for support information\Lime 10pH. Matrix 10%.sav |
|  | Active Dataset | DataSet11 |
|  | Filter | <none> |
|  | Weight | <none> |
|  | Split File | <none> |
|  | N of Rows in Working Data File | 12 |
| Missing Value Handling | Definition of Missing | User defined missing values are treated as missing. |
|  | Cases Used | Statistics for each analysis are based on the cases with no missing or out-of-range data for any variable in the analysis. |
| Syntax | | T-TEST GROUPS=Mixture(1 2)  /MISSING=ANALYSIS  /VARIABLES=pH  /CRITERIA=CI(.95). |
| Resources | Processor Time | 00:00:00.00 |
|  | Elapsed Time | 00:00:00.01 |

[DataSet11] C:\Users\dds12\Dropbox\BUCKET PAPER\Bucket paper SPSS + Excell\PH for support information\Lime 10pH. Matrix 10%.sav

| **Group Statistics** | | | | | |
| --- | --- | --- | --- | --- | --- |
|  | Mixture | N | Mean | Std. Deviation | Std. Error Mean |
| pH | Mixed | 6 | 12.3333 | .39185 | .15997 |
|  | Not mixed | 6 | 12.2633 | .24468 | .09989 |

| **Independent Samples Test** | | | | | | | | | | |
| --- | --- | --- | --- | --- | --- | --- | --- | --- | --- | --- |
|  | | Levene's Test for Equality of Variances | | t-test for Equality of Means | | | | | | |
|  |  | F | Sig. | t | df | Sig. (2-tailed) | Mean Difference | Std. Error Difference | 95% Confidence Interval of the Difference | |
|  |  |  |  |  |  |  |  |  | Lower | Upper |
| pH | Equal variances assumed | 3.137 | .107 | .371 | 10 | .718 | .07000 | .18860 | -.35022 | .49022 |
|  | Equal variances not assumed |  |  | .371 | 8.384 | .720 | .07000 | .18860 | -.36146 | .50146 |

**T-Test**

| **Notes** | | |
| --- | --- | --- |
| Output Created | | 23-JUN-2017 15:27:42 |
| Comments | |  |
| Input | Data | C:\Users\dds12\Dropbox\BUCKET PAPER\Bucket paper SPSS + Excell\PH for support information\Lime 10pH. Matrix 20%.sav |
|  | Active Dataset | DataSet12 |
|  | Filter | <none> |
|  | Weight | <none> |
|  | Split File | <none> |
|  | N of Rows in Working Data File | 15 |
| Missing Value Handling | Definition of Missing | User defined missing values are treated as missing. |
|  | Cases Used | Statistics for each analysis are based on the cases with no missing or out-of-range data for any variable in the analysis. |
| Syntax | | T-TEST GROUPS=Mixture(1 2)  /MISSING=ANALYSIS  /VARIABLES=pH  /CRITERIA=CI(.95). |
| Resources | Processor Time | 00:00:00.00 |
|  | Elapsed Time | 00:00:00.00 |

[DataSet12] C:\Users\dds12\Dropbox\BUCKET PAPER\Bucket paper SPSS + Excell\PH for support information\Lime 10pH. Matrix 20%.sav

| **Group Statistics** | | | | | |
| --- | --- | --- | --- | --- | --- |
|  | Mixture | N | Mean | Std. Deviation | Std. Error Mean |
| pH | Mixed | 6 | 12.6750 | .21078 | .08605 |
|  | Not mixed | 9 | 12.3733 | .38243 | .12748 |

| **Independent Samples Test** | | | | | | | | | | |
| --- | --- | --- | --- | --- | --- | --- | --- | --- | --- | --- |
|  | | Levene's Test for Equality of Variances | | t-test for Equality of Means | | | | | | |
|  |  | F | Sig. | t | df | Sig. (2-tailed) | Mean Difference | Std. Error Difference | 95% Confidence Interval of the Difference | |
|  |  |  |  |  |  |  |  |  | Lower | Upper |
| pH | Equal variances assumed | .502 | .491 | 1.749 | 13 | .104 | .30167 | .17247 | -.07094 | .67427 |
|  | Equal variances not assumed |  |  | 1.961 | 12.725 | .072 | .30167 | .15380 | -.03133 | .63467 |

**T-Test**

| **Notes** | | |
| --- | --- | --- |
| Output Created | | 23-JUN-2017 15:28:24 |
| Comments | |  |
| Input | Data | C:\Users\dds12\Dropbox\BUCKET PAPER\Bucket paper SPSS + Excell\PH for support information\Lime 20pH Matrix 0%.sav |
|  | Active Dataset | DataSet13 |
|  | Filter | <none> |
|  | Weight | <none> |
|  | Split File | <none> |
|  | N of Rows in Working Data File | 12 |
| Missing Value Handling | Definition of Missing | User defined missing values are treated as missing. |
|  | Cases Used | Statistics for each analysis are based on the cases with no missing or out-of-range data for any variable in the analysis. |
| Syntax | | T-TEST GROUPS=Mixture(1 2)  /MISSING=ANALYSIS  /VARIABLES=pH  /CRITERIA=CI(.95). |
| Resources | Processor Time | 00:00:00.00 |
|  | Elapsed Time | 00:00:00.00 |

[DataSet13] C:\Users\dds12\Dropbox\BUCKET PAPER\Bucket paper SPSS + Excell\PH for support information\Lime 20pH Matrix 0%.sav

| **Group Statistics** | | | | | |
| --- | --- | --- | --- | --- | --- |
|  | Mixture | N | Mean | Std. Deviation | Std. Error Mean |
| pH | Mixed | 6 | 12.9842 | .31765 | .12968 |
|  | Not mixed | 6 | 12.8700 | .06197 | .02530 |

| **Independent Samples Test** | | | | | | | | | | |
| --- | --- | --- | --- | --- | --- | --- | --- | --- | --- | --- |
|  | | Levene's Test for Equality of Variances | | t-test for Equality of Means | | | | | | |
|  |  | F | Sig. | t | df | Sig. (2-tailed) | Mean Difference | Std. Error Difference | 95% Confidence Interval of the Difference | |
|  |  |  |  |  |  |  |  |  | Lower | Upper |
| pH | Equal variances assumed | 10.225 | .010 | .864 | 10 | .408 | .11417 | .13213 | -.18023 | .40856 |
|  | Equal variances not assumed |  |  | .864 | 5.380 | .424 | .11417 | .13213 | -.21838 | .44672 |

**T-Test**

| **Notes** | | |
| --- | --- | --- |
| Output Created | | 23-JUN-2017 15:29:34 |
| Comments | |  |
| Input | Data | C:\Users\dds12\Dropbox\BUCKET PAPER\Bucket paper SPSS + Excell\PH for support information\Lime 20pH Matrix 20%.sav |
|  | Active Dataset | DataSet14 |
|  | Filter | <none> |
|  | Weight | <none> |
|  | Split File | <none> |
|  | N of Rows in Working Data File | 12 |
| Missing Value Handling | Definition of Missing | User defined missing values are treated as missing. |
|  | Cases Used | Statistics for each analysis are based on the cases with no missing or out-of-range data for any variable in the analysis. |
| Syntax | | T-TEST GROUPS=Mixture(1 2)  /MISSING=ANALYSIS  /VARIABLES=pH  /CRITERIA=CI(.95). |
| Resources | Processor Time | 00:00:00.00 |
|  | Elapsed Time | 00:00:00.00 |

[DataSet14] C:\Users\dds12\Dropbox\BUCKET PAPER\Bucket paper SPSS + Excell\PH for support information\Lime 20pH Matrix 20%.sav

| **Group Statistics** | | | | | |
| --- | --- | --- | --- | --- | --- |
|  | Mixture | N | Mean | Std. Deviation | Std. Error Mean |
| pH | Mixed | 6 | 13.0200 | .19555 | .07983 |
|  | Not mixed | 6 | 12.5867 | .06713 | .02741 |

| **Independent Samples Test** | | | | | | | | | | |
| --- | --- | --- | --- | --- | --- | --- | --- | --- | --- | --- |
|  | | Levene's Test for Equality of Variances | | t-test for Equality of Means | | | | | | |
|  |  | F | Sig. | t | df | Sig. (2-tailed) | Mean Difference | Std. Error Difference | 95% Confidence Interval of the Difference | |
|  |  |  |  |  |  |  |  |  | Lower | Upper |
| pH | Equal variances assumed | 5.447 | .042 | 5.134 | 10 | .000 | .43333 | .08441 | .24526 | .62140 |
|  | Equal variances not assumed |  |  | 5.134 | 6.162 | .002 | .43333 | .08441 | .22811 | .63856 |

**T-Test**

| **Notes** | | |
| --- | --- | --- |
| Output Created | | 23-JUN-2017 15:30:20 |
| Comments | |  |
| Input | Data | C:\Users\dds12\Dropbox\BUCKET PAPER\Bucket paper SPSS + Excell\PH for support information\Lime 20pH Matrix10%.sav |
|  | Active Dataset | DataSet15 |
|  | Filter | <none> |
|  | Weight | <none> |
|  | Split File | <none> |
|  | N of Rows in Working Data File | 12 |
| Missing Value Handling | Definition of Missing | User defined missing values are treated as missing. |
|  | Cases Used | Statistics for each analysis are based on the cases with no missing or out-of-range data for any variable in the analysis. |
| Syntax | | T-TEST GROUPS=Mixture(1 2)  /MISSING=ANALYSIS  /VARIABLES=pH  /CRITERIA=CI(.95). |
| Resources | Processor Time | 00:00:00.00 |
|  | Elapsed Time | 00:00:00.00 |

[DataSet15] C:\Users\dds12\Dropbox\BUCKET PAPER\Bucket paper SPSS + Excell\PH for support information\Lime 20pH Matrix10%.sav

| **Group Statistics** | | | | | |
| --- | --- | --- | --- | --- | --- |
|  | Mixture | N | Mean | Std. Deviation | Std. Error Mean |
| pH | Mixed | 6 | 12.6350 | .23313 | .09518 |
|  | Not mixed | 6 | 12.6317 | .16916 | .06906 |

| **Independent Samples Test** | | | | | | | | | | |
| --- | --- | --- | --- | --- | --- | --- | --- | --- | --- | --- |
|  | | Levene's Test for Equality of Variances | | t-test for Equality of Means | | | | | | |
|  |  | F | Sig. | t | df | Sig. (2-tailed) | Mean Difference | Std. Error Difference | 95% Confidence Interval of the Difference | |
|  |  |  |  |  |  |  |  |  | Lower | Upper |
| pH | Equal variances assumed | .992 | .343 | .028 | 10 | .978 | .00333 | .11759 | -.25868 | .26534 |
|  | Equal variances not assumed |  |  | .028 | 9.122 | .978 | .00333 | .11759 | -.26213 | .26880 |

**T-Test**

| **Notes** | | |
| --- | --- | --- |
| Output Created | | 23-JUN-2017 15:30:55 |
| Comments | |  |
| Input | Data | C:\Users\dds12\Dropbox\BUCKET PAPER\Bucket paper SPSS + Excell\PH for support information\Lime 30pH Matrix 0%.sav |
|  | Active Dataset | DataSet16 |
|  | Filter | <none> |
|  | Weight | <none> |
|  | Split File | <none> |
|  | N of Rows in Working Data File | 13 |
| Missing Value Handling | Definition of Missing | User defined missing values are treated as missing. |
|  | Cases Used | Statistics for each analysis are based on the cases with no missing or out-of-range data for any variable in the analysis. |
| Syntax | | T-TEST GROUPS=Mixture(1 2)  /MISSING=ANALYSIS  /VARIABLES=pH  /CRITERIA=CI(.95). |
| Resources | Processor Time | 00:00:00.00 |
|  | Elapsed Time | 00:00:00.00 |

[DataSet16] C:\Users\dds12\Dropbox\BUCKET PAPER\Bucket paper SPSS + Excell\PH for support information\Lime 30pH Matrix 0%.sav

| **Group Statistics** | | | | | |
| --- | --- | --- | --- | --- | --- |
|  | Mixture | N | Mean | Std. Deviation | Std. Error Mean |
| pH | Mixed | 6 | 12.9933 | .30826 | .12585 |
|  | Not mixed | 7 | 12.9057 | .06321 | .02389 |

| **Independent Samples Test** | | | | | | | | | | |
| --- | --- | --- | --- | --- | --- | --- | --- | --- | --- | --- |
|  | | Levene's Test for Equality of Variances | | t-test for Equality of Means | | | | | | |
|  |  | F | Sig. | t | df | Sig. (2-tailed) | Mean Difference | Std. Error Difference | 95% Confidence Interval of the Difference | |
|  |  |  |  |  |  |  |  |  | Lower | Upper |
| pH | Equal variances assumed | 12.542 | .005 | .739 | 11 | .475 | .08762 | .11851 | -.17321 | .34845 |
|  | Equal variances not assumed |  |  | .684 | 5.361 | .522 | .08762 | .12810 | -.23510 | .41034 |

**T-Test**

| **Notes** | | |
| --- | --- | --- |
| Output Created | | 23-JUN-2017 15:31:25 |
| Comments | |  |
| Input | Data | C:\Users\dds12\Dropbox\BUCKET PAPER\Bucket paper SPSS + Excell\PH for support information\Lime 30pH Matrix 10%.sav |
|  | Active Dataset | DataSet17 |
|  | Filter | <none> |
|  | Weight | <none> |
|  | Split File | <none> |
|  | N of Rows in Working Data File | 12 |
| Missing Value Handling | Definition of Missing | User defined missing values are treated as missing. |
|  | Cases Used | Statistics for each analysis are based on the cases with no missing or out-of-range data for any variable in the analysis. |
| Syntax | | T-TEST GROUPS=Mixture(1 2)  /MISSING=ANALYSIS  /VARIABLES=pH  /CRITERIA=CI(.95). |
| Resources | Processor Time | 00:00:00.02 |
|  | Elapsed Time | 00:00:00.00 |

[DataSet17] C:\Users\dds12\Dropbox\BUCKET PAPER\Bucket paper SPSS + Excell\PH for support information\Lime 30pH Matrix 10%.sav

| **Group Statistics** | | | | | |
| --- | --- | --- | --- | --- | --- |
|  | Mixture | N | Mean | Std. Deviation | Std. Error Mean |
| pH | Mixed | 6 | 12.7917 | .12057 | .04922 |
|  | Not mixed | 6 | 12.7983 | .05913 | .02414 |

| **Independent Samples Test** | | | | | | | | | | |
| --- | --- | --- | --- | --- | --- | --- | --- | --- | --- | --- |
|  | | Levene's Test for Equality of Variances | | t-test for Equality of Means | | | | | | |
|  |  | F | Sig. | t | df | Sig. (2-tailed) | Mean Difference | Std. Error Difference | 95% Confidence Interval of the Difference | |
|  |  |  |  |  |  |  |  |  | Lower | Upper |
| pH | Equal variances assumed | 1.689 | .223 | -.122 | 10 | .906 | -.00667 | .05482 | -.12882 | .11549 |
|  | Equal variances not assumed |  |  | -.122 | 7.274 | .907 | -.00667 | .05482 | -.13532 | .12199 |

**T-Test**

| **Notes** | | |
| --- | --- | --- |
| Output Created | | 23-JUN-2017 15:31:52 |
| Comments | |  |
| Input | Data | C:\Users\dds12\Dropbox\BUCKET PAPER\Bucket paper SPSS + Excell\PH for support information\Lime 30pH Matrix 20%.sav |
|  | Active Dataset | DataSet18 |
|  | Filter | <none> |
|  | Weight | <none> |
|  | Split File | <none> |
|  | N of Rows in Working Data File | 12 |
| Missing Value Handling | Definition of Missing | User defined missing values are treated as missing. |
|  | Cases Used | Statistics for each analysis are based on the cases with no missing or out-of-range data for any variable in the analysis. |
| Syntax | | T-TEST GROUPS=Mixture(1 2)  /MISSING=ANALYSIS  /VARIABLES=pH  /CRITERIA=CI(.95). |
| Resources | Processor Time | 00:00:00.00 |
|  | Elapsed Time | 00:00:00.00 |

[DataSet18] C:\Users\dds12\Dropbox\BUCKET PAPER\Bucket paper SPSS + Excell\PH for support information\Lime 30pH Matrix 20%.sav

| **Group Statistics** | | | | | |
| --- | --- | --- | --- | --- | --- |
|  | Mixture | N | Mean | Std. Deviation | Std. Error Mean |
| pH | Mixed | 6 | 12.9950 | .11432 | .04667 |
|  | Not mixed | 6 | 12.8333 | .12785 | .05220 |

| **Independent Samples Test** | | | | | | | | | | |
| --- | --- | --- | --- | --- | --- | --- | --- | --- | --- | --- |
|  | | Levene's Test for Equality of Variances | | t-test for Equality of Means | | | | | | |
|  |  | F | Sig. | t | df | Sig. (2-tailed) | Mean Difference | Std. Error Difference | 95% Confidence Interval of the Difference | |
|  |  |  |  |  |  |  |  |  | Lower | Upper |
| pH | Equal variances assumed | .667 | .433 | 2.309 | 10 | .044 | .16167 | .07002 | .00565 | .31768 |
|  | Equal variances not assumed |  |  | 2.309 | 9.877 | .044 | .16167 | .07002 | .00539 | .31794 |
