# Supplementary material for: Assessment of recommended approaches for containment and safe handling of human excreta in emergency settings
Source: PLoS One. 2018 Jul 26;13(7):e0201344. doi: 10.1371/journal.pone.0201344 (PMC6062132; doi:10.1371/journal.pone.0201344)
Supplement: S1 Table — (DOCX) [file pone.0201344.s008.docx]

**S1 Table**. **Summary of the recommendations for safe handling, containment and removal of human excreta in Ebola Treatment Centres**

| **Organization** | **Disinfectant** | **Controlled excretions (into a bucket)** |
| --- | --- | --- |
| **MSF, 2008** | "HTH is the recommended chlorine product" (MSF, 2008; pg 102) | "Collect waste in a bucket with 2cm of 0.5% chlorine solution. Add 0.5% chlorine with a cup sufficient to cover the waste in a bucket for **15 minutes**" (MSF, 2008; pg 105) |
| **WHO, 2014** | No specific chlorine type stated. | For any environmental surface contaminated with excretions:  Clean the surface with soap. Disinfect with 0.5% chlorine. Let stand **10 minutes**. Remove with towel.  “Environmental surfaces or objects contaminated with blood, other body fluids, secretions or excretions should be cleaned and disinfected as soon as possible using standard hospital detergents / disinfectants.” (WHO, 2014; pg. 10) |
| **CDC, 2015** | No specific chlorine type stated. | "Clean prior to disinfecting” (CDC, 2015b)"Keep the bucket closed. Carefully pour enough of the 0.5% chlorine solution into the bucket to cover the contents. Let the solution stay for **30 minutes**" (CDC, 2015a) |
| **WHO, 2014** | Hydrated lime | “Assuming a 10-litre covered bucket, first add approximately 600 ml (three cups) of a 10% (i.e., 100 g of lime powder in 1 litre of water) slurry (suspension) of hydrated (slaked) lime to the bucket. Then, carefully add the excreta from the bedpan into the bucket, leaving sufficient space in the bucket to add safely at least an additional 400 ml (two cups) of lime slurry. Rinse and disinfect the bedpan as described above. The final product should continue to be treated with caution and be carefully disposed of in a toilet or latrine by a person wearing full PPE” |
