# Supplementary material for: Assessment of recommended approaches for containment and safe handling of human excreta in emergency settings
Source: PLoS One. 2018 Jul 26;13(7):e0201344. doi: 10.1371/journal.pone.0201344 (PMC6062132; doi:10.1371/journal.pone.0201344)
Supplement: S2 Table — (DOCX) [file pone.0201344.s009.docx]

**S2 Table.** **Average levels (median and geometric mean) of surviving indicator organisms according to each approach.** Pooled samples (n=216; 6 disinfectants*2 contact times* 2 mixtures* 3 excreta matrices* 3 repetitions)

| **Disinfectants** |  | **Final microorganism count , CFU or PFU ml^-1^** | | | | |
| --- | --- | --- | --- | --- | --- | --- |
|  | **average** | **FC** | **IE** | **SOMPH** | **F+PH** | **GB124PH** |
| **HTH** | **M** | 413 | 453 | 113 | 1,850 | 75,250 |
|  | **GM** | 92 | 160 | 63 | 303 | 2,238 |
| **NaDCC** | **M** | 145 | 505 | 55 | 669 | 72,625 |
|  | **GM** | 60 | 127 | 39 | 190 | 2,062 |
| **Bleach** | **M** | 338 | 1,610 | 150 | 1,445 | 40,375 |
|  | **GM** | 119 | 249 | 66 | 243 | 2,418 |
| **Lime 10%** | **M** | 1* | 83 | 1,538 | 1* | 1* |
|  | **GM** | 5* | 58 | 259 | 3* | 4* |
| **Lime 20%** | **M** | 1* | 5* | 313 | 1* | 1* |
|  | **GM** | 5* | 16 | 102 | 2* | 3* |
| **Lime 30%** | **M** | 1* | 1* | 133 | 1* | 1* |
|  | **GM** | 3* | 10 | 66 | 1* | 2* |

* = Substitute for detection limits, 10 PFU or CFU.
