# Supplementary material for: Assessment of recommended approaches for containment and safe handling of human excreta in emergency settings
Source: PLoS One. 2018 Jul 26;13(7):e0201344. doi: 10.1371/journal.pone.0201344 (PMC6062132; doi:10.1371/journal.pone.0201344)
Supplement: S3 Table — (DOCX) [file pone.0201344.s010.docx]

**S3 Table. Approaches efficacy (median log reduction) according to contact time**

| **Disinfectant** | **Ct (minutes)** | **samples** | **FC** | **IE** | **SOMPH** | **F^+^ PH** | **GB124PH** |
| --- | --- | --- | --- | --- | --- | --- | --- |
| **HTH** | 10 | 18 | 2.56 | 1.77 | 2.44 | 1.62 | 0.65 |
|  | 30 | 18 | 2.55 | 2.10 | 2.66 | 1.96 | 0.69 |
| **NaDCC** | 10 | 18 | 2.70 | 1.84 | 2.67 | 2.36 | 0.72 |
|  | 30 | 18 | 2.95 | 2.51 | 3.51 | 2.26 | 0.75 |
| **Bleach** | 10 | 18 | 2.14 | 1.34 | 2.24 | 1.76 | 0.64 |
|  | 30 | 18 | 2.21 | 1.84 | 2.38 | 1.92 | 0.62 |
| **Lime 10%** | 10 | 18 | 4.59 | 3.02 | 1.67 | 5.13 | 5.14 |
|  | 30 | 18 | 4.55 | 3.11 | 1.89 | 5.13 | 5.53 |
| **Lime 20%** | 10 | 18 | 4.67 | 3.96 | 2.24 | 5.13 | 5.14 |
|  | 30 | 18 | 4.54 | 3.91 | 2.25 | 5.13 | 5.53 |
| **Lime 30%** | 10 | 18 | 4.82 | 4.08 | 2.85 | 5.13 | 5.14 |
|  | 30 | 18 | 4.72 | 4.25 | 2.70 | 5.13 | 5.53 |
