# Supplementary material for: Assessment of recommended approaches for containment and safe handling of human excreta in emergency settings
Source: PLoS One. 2018 Jul 26;13(7):e0201344. doi: 10.1371/journal.pone.0201344 (PMC6062132; doi:10.1371/journal.pone.0201344)
Supplement: S4 Table — (DOCX) [file pone.0201344.s011.docx]

**S4 Table.** **Approaches efficacy (median log reduction) according to mixing regime**

| **Disinfectant** | **Treatment** | **samples** | **FC** | **IE** | **SOMPH** | **F^+^ PH** | **GB124PH** |
| --- | --- | --- | --- | --- | --- | --- | --- |
| **HTH** | Mixed | 18 | 2.47 | 1.99 | 2.43 | 1.71 | 0.67 |
|  | Non-mixed | 18 | 2.65 | 1.93 | 2.93 | 1.77 | 0.69 |
| **NaDCC** | Mixed | 18 | 2.90 | 2.47 | 2.81 | 2.40 | 0.71 |
|  | Non-mixed | 18 | 2.78 | 1.92 | 3.24 | 2.21 | 0.76 |
| **Bleach** | Mixed | 18 | 2.19 | 1.40 | 2.25 | 1.76 | 0.59 |
|  | Non-mixed | 18 | 2.17 | 1.61 | 3.66 | 1.90 | 0.68 |
| **Lime 10%** | Mixed | 18 | 4.66 | 3.14 | 1.88 | **5.52*** | 5.53 |
|  | Non-mixed | 18 | 4.47 | 2.46 | 1.43 | 4.99 | 5.14 |
| **Lime 20%** | Mixed | 18 | **4.72*** | 3.97 | 2.36 | **5.52*** | 5.44 |
|  | Non-mixed | 18 | 4.38 | 3.55 | 1.85 | 4.99 | 5.14 |
| **Lime 30%** | Mixed | 18 | **4.83*** | 4.25 | 2.97 | **5.52*** | 5.41 |
|  | Non-mixed | 18 | 4.41 | 3.94 | 1.80 | 4.99 | 5.40 |

***** = Log reduction values in bold were significantly greater (p < 0.05) than the other Log reductions observed within the mixing regimes
